# Interaction of MRI Contrast Agent [Gd(DOTA)]^−^ with Lipid Membranes: A Molecular Dynamics Study

**DOI:** 10.1021/acs.inorgchem.4c00972

**Published:** 2024-05-25

**Authors:** Alexandre C. Oliveira, Hugo A. L. Filipe, Carlos F.G.C. Geraldes, Gregory A. Voth, Maria João Moreno, Luís M. S. Loura

**Affiliations:** † Coimbra Chemistry Centre, Institute of Molecular Sciences (CQC-IMS), 3004-535 Coimbra, Portugal; ‡ Department of Chemistry, University of Coimbra, 3004-535 Coimbra, Portugal; § CPIRN-IPG—Center of Potential and Innovation of Natural Resources, Polytechnic Institute of Guarda, 6300-559 Guarda, Portugal; ∥ Department of Life Sciences, University of Coimbra, Calçada Martim de Freitas, 3000-393 Coimbra, Portugal; ⊥ CIBIT/ICNAS - Instituto de Ciências Nucleares Aplicadas à Saúde, Pólo das Ciências da Saúde, Azinhaga de Santa Comba, 3000-548 Coimbra, Portugal; # Department of Chemistry, Chicago Center for Theoretical Chemistry, James Franck Institute, and Institute for Biophysical Dynamics, University of Chicago, Chicago, Illinois 60637, United States; ▽ Faculty of Pharmacy, University of Coimbra, 3000-548 Coimbra, Portugal; ○ CNC−Center for Neuroscience and Cell Biology, University of Coimbra, 3004-517 Coimbra, Portugal

## Abstract

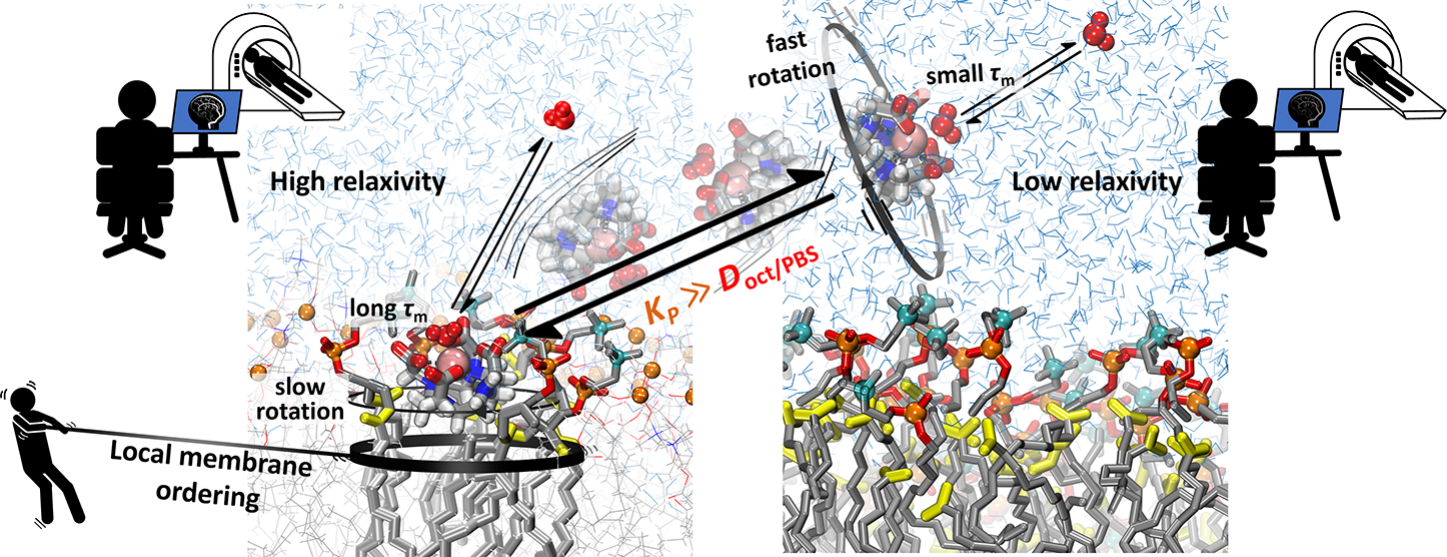

Contrast agents are important imaging probes in clinical MRI, allowing the identification of anatomic changes that otherwise would not be possible. Intensive research on the development of new contrast agents is being made to image specific pathological markers or sense local biochemical changes. The most widely used MRI contrast agents are based on gadolinium(III) complexes. Due to their very high charge density, they have low permeability through tight biological barriers such as the blood-brain barrier, hampering their application in the diagnosis of neurological disorders. In this study, we explore the interaction between the widely used contrast agent [Gd(DOTA)]^−^ (Dotarem) and POPC lipid bilayers by means of molecular dynamics simulations. This metal complex is a standard reference where several chemical modifications have been introduced to improve key properties such as bioavailability and targeting. The simulations unveil detailed insights into the agent’s interaction with the lipid bilayer, offering perspectives beyond experimental methods. Various properties, including the impact on global and local bilayer properties, were analyzed. As expected, the results indicate a low partition coefficient (*K*_P_) and high permeation barrier for this reference compound. Nevertheless, favorable interactions are established with the membrane leading to moderately long residence times. While coordination of one inner-sphere water molecule is maintained for the membrane-associated chelate, the physical-chemical attributes of [Gd(DOTA)]^−^ as a MRI contrast agent are affected. Namely, increases in the rotational correlation times and in the residence time of the inner-sphere water are observed, with the former expected to significantly increase the water proton relaxivity. This work establishes a reference framework for the use of simulations to guide the rational design of new contrast agents with improved relaxivity and bioavailability and for the development of liposome-based formulations for use as imaging probes or theranostic agents.

## Abstract 2

All-atom molecular dynamics simulations of MRI contrast agent [Gd(DOTA)]^−^ (Dotarem) are employed to thoroughly characterize its interactions with POPC lipid membranes. Despite its hydrophilicity, [Gd(DOTA)]^−^ inserts modestly into the lipid bilayer, inducing local ordering and slight overall disordering. While inserted, [Gd(DOTA)]^−^ maintains coordination of one inner-sphere water molecule. Therefore, [Gd(DOTA)]^−^ acts as an efficient MRI contrast agent even when associated with the membrane, albeit with changed physical-chemical properties, which are addressed here in detail.

## 1 Introduction

Medical imaging is of utmost importance in the medical field, providing a tool for the noninvasive diagnosis of pathologies. The variety of available modalities is accompanied by the diversity of imaging probes developed to detect specific physiological or pathological markers. In this regard, lanthanide complexes have a central role, as different lanthanides can be used in different modalities. For example, Eu(III) complexes can be used in optical imaging,^1^^153^Sm(III) in SPECT^2^ and Gd(III) in magnetic resonance imaging (MRI).^3,4^

**Figure 1:**
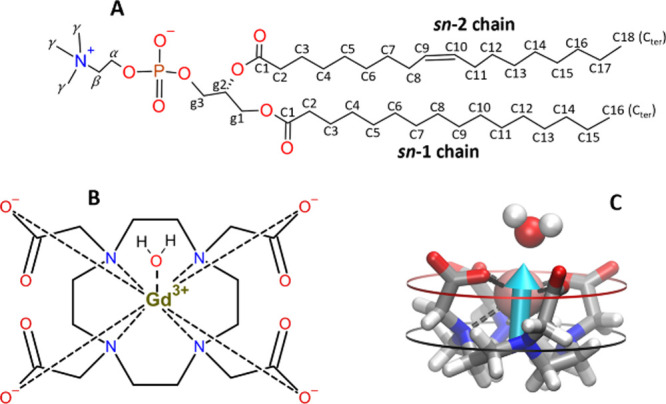
Illustration of chemical structure of 1-palmitoyl-2-oleoyl-*sn*-glycero-3-phosphocholine (POPC) with the indication of the heavy atoms labels relevant to this work (A). Structure of the metal complex [Gd(DOTA)(H_2_O)]^−^ (B). 3D representation of the metal complex with the indication of the hydrophilic part of the metal complex (red circle) that corresponds to the carboxylic groups (COO^–^) and the hydrophobic part (black circle) that corresponds to the tetraaza macrocyclic ring. The arrow represents the vector used to determine the metal complex orientation (C).

The MRI technique is based on the environmental dependence of water proton relaxivity, leading to high-resolution images. There are different types of physical phenomena that can be explored in MRI. However, the *T*_1_-weighted images based on the spin–lattice relaxation time (*T*_1_) of the water protons constitute the relevant modality for Gd^3+^-based contrast agents (GBCAs), such as [Gd(DOTA)]^−^ (Dotarem; Figure 1). Gd^3+^-based complexes accelerate the relaxation process of the water protons in their vicinity, significantly decreasing *T*_1_ and producing a hyperintense signal in the MRI image. The rationalization of the nuclear relaxation effect produced by a paramagnetic Gd^3+^-based contrast agent in a dilute solution is based on the classical Solomon–Bloembergen–Morgan (SBM) and Swift–Connick equations. The paramagnetic relaxation process of the water protons results from the dipole–dipole interactions between the nuclear spins and the fluctuating local magnetic field originated by the Gd^3+^ unpaired electron spins, the value of which decreases rapidly with distance. It is usually split into the inner-sphere, second-sphere, and outer-sphere contributions, which depend on the chemical interactions that bring the water protons close to the Gd^3+^ ion and transmit the paramagnetic effect into the bulk solvent. The inner-sphere contribution reflects the exchange of the water molecule(s) bound in the Gd^3+^ first coordination sphere with the bulk water molecules, which depend on the number of coordinated water molecules (*q*), the distance between the water protons and the electron spin of Gd^3+^ (*r*_GdH_), the residence time of the inner-sphere water molecule(s) (τ_m_), the rotational correlation time (τ_R_) of the metal complex, and the correlation times (τ_ci_, with *i* = 1,2). Higher *q* and shorter *r*_GdH_ values increase the proton relaxivity. The inverse correlation times characteristic of the relaxation process are given by the sum of the inverses of the characteristic times of the following three processes: 1/τ_ci_ = 1/τ_m_ + 1/τ_R_ + 1/*T*_*i*__e_ (*i* = 1,2), where *T*_1e_ and *T*_2e_ are the longitudinal and transverse electron spin relaxation times of Gd^3+^. The fastest process will be the dominant term in the correlation times and consequently in the relaxation process. The rotational term is usually dominant for small GBCAs. Therefore, by slowing down the rotation, the relaxation of the water protons increases significantly. The residence lifetime of the water molecule(s) in the inner-sphere is a parameter that influences the relaxation process in different ways. For that reason, τ_m_ cannot be too short since it will not transfer the paramagnetic effect to the inner-sphere water molecule(s) and cannot be too long since it will not allow the transfer of the paramagnetic effect to the bulk water protons. The outer-sphere term is based on the random translational diffusion of the water molecules in the vicinity of the metal ion. For certain GBCAs, solvent water molecules may remain close to the metal complex for relatively long times, without binding directly in the first coordination sphere. This is achieved through interactions with other groups of the GBCA, namely hydrogen bonds to ligands’ carboxylate or phosphonate groups. The contribution of the second-sphere to the overall relaxivity of the GBCA is calculated using the same formalism used for the inner-sphere, see Appendix S.I.4 in the Supporting Information for details.^5−7^

[Gd(DOTA)]^−^ is one of the most important commercially available MRI contrast agents. It is highly hydrophilic (reported log *D*_oct/PBS_ = −4.16)^8^ and therefore expected to interact with biomembranes very weakly. Its high hydrophilicity is due to the combination of high local charged groups and bulky geometry^9^ that makes the lanthanide complexes unlikely to permeate biomembranes, reducing their availability to target tissues protected by tight endothelial cells such as the case of the Blood-Brain-Barrier (BBB). In fact, this metal complex is used to evaluate BBB disruption.^10^ Nevertheless, the very high stability of this metal complex, both thermodynamic and kinetic, ensures its safety use in clinical settings. For this reason, the scientific community uses this chelate as a standard reference from which several chemical modifications are made. These modifications are made to improve several properties such as bioavailability, targeting, and relaxivity. Additionally, these modified metal complexes derived from [Gd(DOTA)]^−^ or other similar macrocyclic metal complexes have also been explored in liposome-based formulations to be employed as novel imaging agents with elevated relaxivity or as a theranostic agents.^6,11−13^

In general, the ability of molecules to partition and permeate through biomembranes is a major key factor in their pharmacokinetics profile, its characterization being of fundamental importance for new imaging probes or drugs. This determines the concentration of the active compound in the target tissue. Examples span from the ability of oral administrated drugs to be absorbed by the digestive tract, to the ability of drugs to cross the BBB (e.g., antidepressants) or to cross the cell membrane to reach intracellular targets.^14,15^ For these reasons, several experimental and computational methods have emerged to predict solute partition to and permeation through membranes. Most experimental methods are laborious and expensive, presenting several drawbacks, starting with the need to synthesize the new compounds. For these reasons, computational methods have emerged as powerful tools, that give qualitative and semiquantitative indications on the interaction and permeability through lipid bilayers at a molecular scale. The main method involves Molecular Dynamics simulations (MD) using molecular mechanics force fields, since it is computationally prohibitive to employ ab initio methods combined with MD for large systems. Much effort has been made to improve the predictability of computational simulations, with the improvement of the force fields^16^ and in the development of new sampling techniques.^17^ Improvements in hardware performance have helped extend the time scales available for MD simulations, currently making a 1–2 μs simulation of a system with 10^5^ particles a relatively common practice.

Modeling lanthanide complexes by molecular mechanics correctly is a challenging task, since most of the common force fields do not have parameters for this kind of compound. In our previous work, [Gd(DOTA)]^−^ was successfully parametrized.^18^ Several physical-chemical properties were determined, including the most relevant ones for its efficiency as a contrast agent (*q*, τ_m_, and τ_R_). This was the first work where τ_m_ was obtained by direct registration of the dissociative events.^18^

Although there are some computational studies where lanthanide complexes were successfully modeled, there are only very few works where MD simulations were used to study the interaction of lanthanide complexes with biomolecules,^19−26^ including lipid membranes.^19,21,23,25^ In particular, little is known about the interaction of contrast agents with membranes and how the properties relevant to MRI are affected while inserted in a lipid membrane. As far as we know, the work closest to that objective was carried out by Isabettini et al., who tried to understand how the DMPE-[Tm(DTPA)] complex affects the magnetic susceptibility (Δ*χ*) of a bicelle.^21^

In the present work, we performed MD simulations to get insights on the interaction of the contrast agent [Gd(DOTA)]^−^ with lipid bilayers. 1-Palmitoyl-2-oleoyl-*sn*-glycero-3-phosphocholine (POPC) was chosen as the component of the lipid bilayer, since it is a very common phospholipid in the eukaryotic plasma membrane.^27−29^ The [Gd(DOTA)]^−^ in a square antiprismatic geometry (SAP) was the stereoisomer simulated given that it is the predominant conformation in aqueous solution (>80% of the population).^30^ To ensure compatibility with the [Gd(DOTA)]^−^ force field, we selected Slipids as the lipid force field.^31−33^ It is well-known that the quality of the lipid force fields is of major importance in the accuracy of membrane MD simulations. For that reason, the community is actively trying to reproduce more accurately fundamental properties of the lipid bilayers (e.g., area/lipid, proton order parameters, and transition temperature).^16^ To this purpose, we first analyze the properties of the lipid force field in a pure lipid bilayer system, since we verified some discrepancies between the old^31,32^ and new^33^ versions of the Slipids force field. Subsequently, in a system with [Gd(DOTA)]^−^ complexes, the free energy profile along the *z* distance between the centers of mass (COM) of [Gd(DOTA)]^−^ and the membrane is calculated, enabling the determination of its partition coefficient to the membrane. Additionally, several properties are analyzed, namely the orientation and equilibrium position while the metal complex is inserted in the membrane, as well as the rotational correlation time and the lifetime of the inner sphere water while the metal complex is located inside or outside the membrane. We also address the effect of [Gd(DOTA)]^−^ on the global and local lipid properties and the interactions that [Gd(DOTA)]^−^ complexes establish with relevant lipid functional groups. With this work, we get clues on how the standard contrast agent [Gd(DOTA)]^−^ interacts with lipid bilayers and how the properties that influence the relaxivity in the presence of a contrast agent are altered while inserted in the membrane.

## 2 Experimental Section

The MD simulations and some analyses were done with GROMACS version 2019 and 2020.^34−36^ Other analyses were done with in-house python coding and with the NMRlipids project code for the determination of the proton order parameters^37,38^ that uses the MDAnalysis module.^39^ Additional python packages were also used namely numpy,^40^ SciPy,^41^ and matplotlib.^42^ Visualization was done with VMD.^43^

The parametrization of the contrast agent [Gd(DOTA)]^−^ in the SAP conformation was obtained from our previous work.^18^ This parametrization is compatible with the General Amber Force Field (GAFF) and uses several parameters from this force field.^44^ The lipid was modeled with the new^33^ and old version of Slipids force field,^31,32^ which is compatible with GAFF. The authors of this force field used several parameters from the CHARMM36 force field to parametrize theirs.^45^ The original TIP3P model was used for the water, where the Lennard-Jones parameters for the hydrogen atoms are equal to zero.^46,47^ Additionally, sodium ion parameters were obtained from Amber force fields, such as GAFF.^44^

The membrane was built with the MemGen tool with 200 POPC molecules and 75 waters per lipid.^48^ To ensure the correct equilibration of the membrane, we started with a minimization step with the steepest descent algorithm, followed by 100 ps *NVT* and 100 ps *NPT* equilibration runs with an integration step of 1 fs. The final step was a production run of 200 ns with a 2 fs integration step at 300 K. These simulations were used to validate and compare the new^33^ and old version of the Slipids Force Field.^31,32^ After the selection of the lipid force field and the cutoff scheme, the corresponding last coordinates of that system were used as the starting structure for an additional run of 1 μs at 310.15 K.

Models of membrane with [Gd(DOTA)]^−^ complexes were built after the correct equilibration of the membrane. In that system, 4 [Gd(DOTA)]^−^ complexes were placed at different positions in each replica. Sodium ions were added to neutralize the systems. Three replicates were simulated with the [Gd(DOTA)]^−^ complexes initially placed in water, while three other replicates had [Gd(DOTA)]^−^ complexes initially inserted in the membrane. For the latter, placement of the [Gd(DOTA)]^−^ complexes was done through steered MD by pulling the [Gd(DOTA)]^−^ complexes from water to the membrane COM. For each replica, besides the different position of the metal complexes in the system, the initial velocities for each particle were always randomly generated, according to a Maxwell–Boltzmann distribution at 310.15 K. Before the production run, an equilibration protocol identical to that described above for the initial equilibration of the POPC membrane was applied.

All the production runs in this work were done in *NPT* conditions with a 2 fs integration step. Periodic boundary conditions were applied in all directions. The electrostatic interactions were modeled with the PME algorithm^49^ with different cutoff radii (*r* = 1.0, 1.2, and 1.4 nm). The van der Waals interactions cutoff was done with a truncation also at different cutoff radii (*r* = 1.0, 1.2, and 1.4 nm, see section 3.1 for more information). The temperature was kept constant at the desired value with the Nose-Hoover algorithm.^50,51^ For the system with only POPC and water, each component was thermalized independently. For the system with metal complexes included, water, ions, and [Gd(DOTA)]^−^ complexes were thermalized together, and a separate thermostat was used for the membrane. The pressure was kept at 1.013 bar using the Parrinello–Rahman algorithm^52^ with semi-isotropic scheme and a compressibility of 4.5 × 10^–5^ bar^–1^. The LINCS algorithm^53^ was used to constrain all the bonds. Dispersion corrections in energy and pressure were applied.

For the analysis of the simulations, the first 20 ns were discarded. The analysis of the properties while the metal complexes were inserted into the membrane was done first by identifying the insertion events. In the partition process of the solute to the membrane, there might exist an adsorption process to the membrane surface before the insertion of the solute into the membrane. To avoid any mixture of processes during the analysis, the starting point of each insertion event was defined as the moment the metal complex remained at least 10 ns within distances less than 3.0 nm from the membrane COM along the normal direction of the bilayer plane (*z* axis). The end of the event was defined as 10 ns before the time the metal complex exceeds 3.0 nm of distance in the *z* axis from the membrane COM. The 10 ns lag time was important to ensure the equilibration of the metal complex in the membrane, in order to calculate equilibrium properties while the metal complex is inserted into the membrane. This was the definition of an insertion event used throughout this work. The calculated properties were averaged over the several events of insertion and desorption with the solute located in the membrane or in water, respectively. For the sake of simplification, some analyses, namely rotational correlational times (τ_R_), local lipid bilayer properties, H-bonds, and the radial distribution function (RDF) of the carbonyl oxygen of POPC around the methylene groups of [Gd(DOTA)]^−^ (sections 3.2), were conducted by averaging the results obtained from one insertion event observed for one specific [Gd(DOTA)]^−^ in each replicate (6 samples in total). For the analysis of the local proton-order parameters, the density maps and the RDF of all the heavy atoms around the COM of [Gd(DOTA)]^−^, the event where the [Gd(DOTA)]^−^ remained inserted in the membrane during the entire simulated time (corresponding to the solute trajectory depicted in Figure S10I) was used.

The uncertainties of the properties calculated from a single run were estimated using the block analysis method first proposed by Flyvbjerg and Petersen^54^ and reinterpreted by others,^55,56^ at 95% confidence interval with a *t*-student distribution (explained in Appendix S.I.1). For the properties obtained with multiple replicas, the confidence interval for the averaged property was also calculated with Student’s *t* distribution at 95% confidence interval. The properties calculated for events occurring with different sampling times were calculated through the weighted mean, with the weights assigned according to the sampling time used to obtain each sampled instance. For the confidence interval of a weighted mean, there are not many formulations in the literature. We use the ratio variance formulated by Cochran^57^ that was first proposed by Endlich et al. as an approximation to the standard error of the weighted mean.^58^ Gatz and Smith demonstrated that this formulation gives results not statistically different from nonparametric bootstrap analysis. This formulation assumes a normal distribution of the data (explained in Appendix S.I.2).^59,60^ When appropriate, the uncertainty was calculated using error propagation equations.^61^

## 3 Results and Discussion

### 3.1 Lipid Force Field

For the studies of the interaction of the [Gd(DOTA)]^−^ complexes with the lipid membrane, we chose the Slipids force field^31,32^ that is compatible with the GAFF^44^ used for the parametrization of the [Gd(DOTA)]^−^ complex. In fact, according to the quality ranking of membrane force fields, defined by the authors of the NMRlipids projects, Slipids is one of the best performing force fields in reproducing experimental properties, especially for POPC membranes.^38^ This force field was also proved to reproduce well the membrane-solute interactions compared to other force fields.^62^ However, some discrepancies between the calculated proton order parameters (-*S*_CH_) of the hydrophilic lipid headgroup of the lipids and experimental values were noted by the authors of the NMRLipids project.^63^ For that reason, an updated version of this force field was published.^33^ However, during this work, we and other authors^64^ noted some discrepancies of this updated version of the force field compared to the original version. For this reason, we start by comparing the old and the new versions of Slipids, to decide which version of the force field is more suited to our purposes.

For the comparison of the updated version of Slipids force field with the old one, we had to set up some MD parameters, since the original version of the force field was developed without the implementation of the cutoff Verlet scheme^65^ in GROMACS. In fact, the MD parameters that were originally chosen are incompatible with more recent versions of GROMACS (later than version 5), that implement the Verlet scheme. Additionally, GPU parallelization, a key aspect to accelerate MD simulations, is only supported in GROMACS with the Verlet scheme. With this new scheme, GROMACS also does not support a van der Waals cut off distance larger than the corresponding Coulomb value, as implemented in the original Slipids force field. In the two first Slipids articles, the developing team modeled the electrostatic interaction with the PME algorithm with a cutoff of 1.4 nm, while the van der Waals interaction cutoff was done with a force-based switch function from 1.4 to 1.5 nm.^31,32^ Despite this, there are several works that used the original Slipids force field with newer versions of GROMACS, employing the cutoff Verlet scheme with the same cutoff radii for the electrostatic and van der Waals interactions. However, no accordance was verified in the choice of the cutoff scheme parameters between those works.^66−71^ For that reason, we tested three sets of cutoff radii for the van der Waals interactions (*r*_VdW_) with hard truncation and for the coulomb interactions (*r*_coulomb_). These cutoff radii were conjugated with the Verlet cutoff scheme and the PME algorithm. Since the Verlet scheme now implemented in GROMACS does not support *r*_VdW_ > *r*_coulomb_, we used the same values for the two radii. The tested radii were 1.0, 1.2, and 1.4 nm. Dispersion correction in energy and pressure were always used. We chose to perform these simulations at 300 K, since the experimental data from Ferreira et al. for the deuterium order parameter of POPC bilayer were obtained at this temperature.^72^ In the work of Kučerka et al., the authors determined the experimental area/lipid and thickness of the POPC bilayer at different temperatures, and by simple linear regression it is possible to obtain those values for the desired temperature (Figure S1).^73^ Analyzing the simulations, upon increasing the cutoff radius from 1.0 to 1.4 nm, we can infer a significant improvement, especially from 1.0 to 1.2 nm, in the area/lipid and in the Luzzati thickness (*D*_B_; calculated from the positions of the membrane where the partial density of water decreases to half the bulk water density),^74^ despite a worsening in the head-to-head distance (*D*_HH_). Concerning the order parameters, a slight improvement was observed from 1.0 to 1.2 nm (MAD, Table 1). Overall, for the cutoff radii of 1.2 and 1.4 nm, similar performances are observed. Since the original and the more recent versions of Slipids force field were developed with higher cutoff radii, we selected *r*_coulomb_ = *r*_VdW_ = 1.4 nm to compare with the updated version of Slipids. The choice of *r*_coulomb_ = *r*_VdW_ = 1.2 nm is an acceptable choice with the advantage of an increase in computational efficiency, but below that radius, a worsening in the membrane properties is expected. As a final point, these authors used, for the calculation of the electrostatic interactions with the PME algorithm, a Fourier spacing of 0.2 nm in the article of the updated version of Slipids, contrasting with 0.1 nm used in ref.^32^ and 0.12 nm in ref.^31^ We opted to maintain the default configuration from GROMACS of 0.12 nm.

**Table 1:** Values of the Area/Lipid Obtained from Figures S2 and S5, Luzzati Thickness (*D*_B_) Obtained from Figures S3 and S6, Head-to-Head Distance (*D*_HH_) and Distance between the N Atoms of the Choline from the Two Monolayers (*D*_N–N_) Obtained from the Data Shown in Figures S4 and S7, Calculated for a POPC Bilayer with the Old^31,32^ and New^33^ Version of Slipids^a^

	Area/lipid (nm^2^)	*D*_B_ (nm)	*D*_HH_ (nm)	*D*_N–N_ (nm)	-*S*_CH_ headgroup MAD	-*S*_CH_*sn*-1 MAD	-*S*_CH_*sn*-2 MAD	-*S*_CH_ overall MAD
New Slipids (*r*_cutoff_ = 1.4 nm)	0.646 (0.005)	3.766	3.772 (0.033)	4.114 (0.028)	0.050	0.032	0.011	0.023
Old Slipids (*r*_cutoff_ = 1.0 nm)	0.658 (0.003)	3.719	3.678 (0.015)	3.983 (0.015)	0.087	0.008	0.012	0.022
Old Slipids (*r*_cutoff_ = 1.2 nm)	0.648 (0.004)	3.782	3.719 (0.024)	4.018 (0.019)	0.083	0.008	0.009	0.019
Old Slipids(*r*_cutoff_ = 1.4 nm)	0.645 (0.007)	3.852	3.725 (0.045)	4.026 (0.041)	0.088	0.008	0.010	0.020
Experimental	0.637	3.935	3.693					

a Average simulation values are given from the simulation with the corresponding maximal amplitudes of the respective 95% confidence intervals in parentheses obtained through the block analysis method (section 2 and Appendix S.I.1). Experimental data were obtained from Kučerka et al.^73^ and used for estimating values for 300 K by linear regression (Figure S1). The mean absolute deviations (MAD) of the order parameters (Figure S8 and Figure 2) were calculated comparing to experimental data from Ferreira et al.^72^

For comparison of the old and new versions of Slipids, we kept the same MD parameters, as described previously. Similar area/lipid values were obtained. However, a worsening of *D*_B_ and *D*_HH_ was obtained for the updated version of Slipids compared to the old one. Concerning the order parameters, we verify that the authors were indeed able to improve them in the lipid headgroup with a significant reduction on the MAD (Table 1). The major forking issue in the g1 prochiral carbon identified by the authors of the NMRLipids project^63^ was resolved in this updated version of Slipids. However, a significant worsening on the order parameters and a splitting of the C2 and C3 carbon atoms of the *sn*-1 tail of POPC were observed for the updated version of Slipids, with an increase in the MAD value (Figure 2 and Table 1). No significant changes were observed on the MAD for the *sn*-2 chain with the forking issue in the C2 and C3 prochiral carbon atoms, identified in the old version by Piggot et al.,^70^ persisting in the updated version. Overall, there is a worsening, with a higher MAD value, of the order parameters of the updated version of Slipids force field compared to the old version. Another discrepancy noted in the density profiles and in the *z* position of the POPC groups along the simulation (Figures S3 and S4) is the difference in the relative positions of the ester groups in the *sn*-1 and *sn*-2 acyl chains. In the old version of Slipids, there is an evidently deeper position of the ester *sn*-1 group compared to its counterpart in the *sn*-2 chain, as expected (Figures S6 and S7). With the updated version of Slipids, an overlap of the positions of the ester of the *sn*-1 and *sn*-2 tail is observed. The rationalization is based on the fact that the glycerol of POPC is not completely parallel to the bilayer plane, with the g3 carbon adopting an upper *z* position compared to g1 and g2. Additionally, the *sn*-1 chain is shorter than its *sn*-2 chain counterpart. Due to the hydrophobic effect, the *sn*-1 ester group is forced to adopt a deeper position compared to the *sn*-2 ester for the *sn*-1 terminal carbon to reach the membrane center. Because of that, the terminal carbon of the *sn*-1 chain is also expected to be in a deeper position than the terminal carbon of the *sn*-2 chain. In the old version of Slipids, in Figure S7C, the deeper position of terminal carbon of the *sn*-1 chain compared to the *sn*-2 chain is evident. However, in the updated version of the force field this is not observed (Figure S4). The worsening of the order parameter for the C2 and C3 of the *sn*-1 chain in the updated version of the Slipids force field is the manifestation of the conformational space in the glycerol region not being well described. For all these reasons, we opted to use the original/old version of POPC Slipids parameters.^31,32^

**Figure 2:**
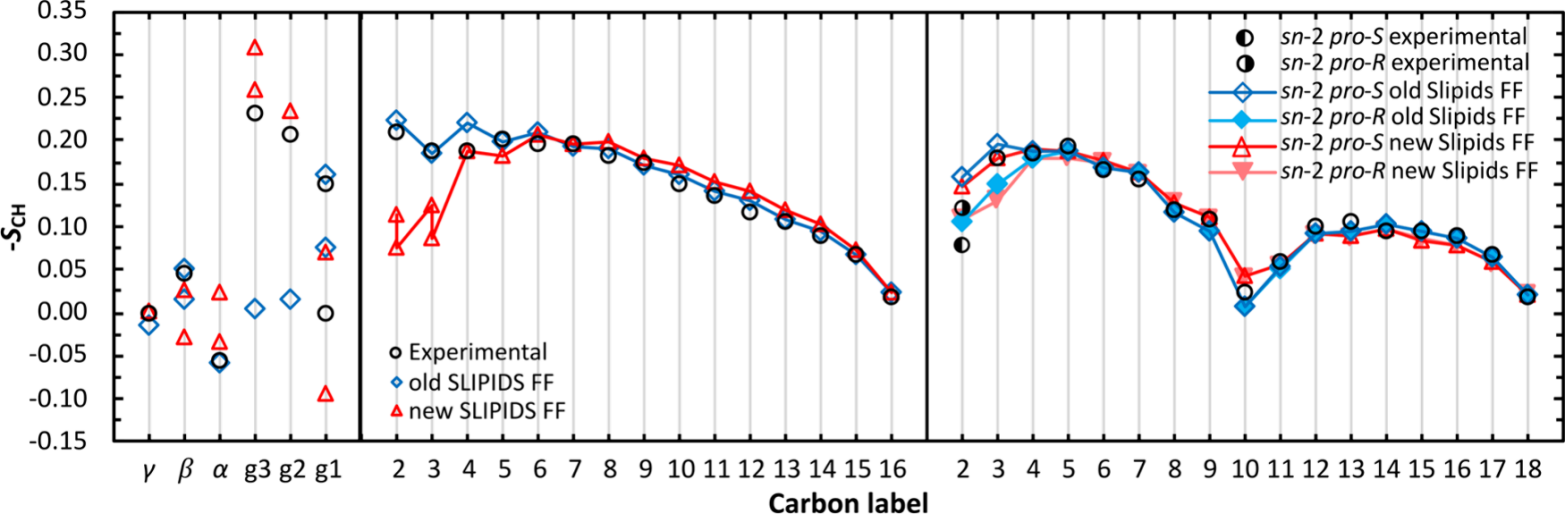
Order parameters (-*S*_CH_) of the headgroup (left) the *sn*-1 tail (middle), and *sn*-2 tail (right) of POPC in the lipid bilayer using the new^33^ and old^31,32^ version of Slipids force field with a cutoff for the electrostatic and van der Waals interactions of 1.4 nm. Also shown are experimental values from ref (72). In the representation of the headgroup and *sn*-1 order parameter, the splitting of the order parameter for the same carbon was only considered when the difference was over 0.02. In the calculation of the *sn*-2 order parameters, the bonds between the prochiral carbon atoms and the *pro*-*S* and *pro*-*R* hydrogen atoms were differentiated in the same way as Piggot et al.^70^

### 3.2 Interaction of [Gd(DOTA)]^−^ with a Lipid Bilayer

#### 3.2.1 Free Energy Profile and Partition to POPC Bilayers

We now turn our attention to the simulations containing [Gd(DOTA)]^−^ complexes in the presence of the POPC bilayer at 310.15 K. In each system, 4 [Gd(DOTA)]^−^ complexes were positioned either in the aqueous medium (Figure S9) or near the membrane COM (Figure S10) as starting positions. As an approximation, in the calculation of each property it was assumed that the four metal complexes in each replica are independent. From Figures S9 and S10, several events of insertion or desorption are observed and in no circumstances any metal complex translocated or even reached the membrane center. Additionally, no evidence of aggregation between [Gd(DOTA)]^−^ was observed. In these figures, hydrophilic and hydrophobic parts of the [Gd(DOTA)]^−^ (see Figure 1 for definition) are also differentiated. When the metal complex inserts in the membrane, a more external position of the hydrophilic part (red line), and conversely a deeper position of the hydrophobic part (gray line) of [Gd(DOTA)]^−^, are observed, the latter penetrating down to the hydrophobic core of the membrane (Figures S9 and S10). From the definition of solute insertion that we have adopted for this work (see Experimental Section), we estimate an average residence time of 240 ± 88 ns for the metal complex inserted in the membrane.

**Figure 3:**
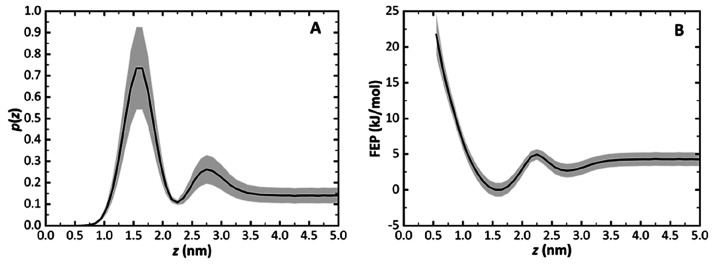
Probability density function as a function of the *z* distance of the [Gd(DOTA)]^−^ complex COM from the membrane COM. The bin sizes are 0.1 nm (A). Free energy profile obtained from the probability density function (B). The gray shade represents the confidence interval at 95% for each bin.

The free energy profile (FEP) along a specific collective variable (CV) gives information about the equilibrium position along that CV and in general the likelihood of the system to explore specific parts of the CV. The FEP for the interaction of the metal complex with the membrane was obtained using as CV the *z* distance between the COM of the metal complex and the COM of the membrane. The ergodicity theory assumes that if the system is simulated for a sufficiently long period of time eventually the whole phase space of the system will be visited. However, the simulation time scales achievable with current hardware are still limited. For that reason, biased simulations have emerged as solutions to overcome this issue.^75^ Nevertheless, in the present work, the simulations were long enough to obtain the FEP between the aqueous medium and the equilibrium position in the membrane. For that purpose, the variation in the free energy can be calculated from the probability density function as^76,77^
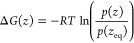
1where Δ*G*(*z*) is the Gibbs free energy along the CV, *R* is the gas constant, *T* is the temperature, *p*(*z*) is the probability density at position *z* along the CV, and *p*(*z*_eq_) is the maximal probability density along the CV. The latter refers to the most probable position, the equilibrium position of the metal complex along the CV. The *p*(*z*) values were obtained using bins of 0.1 nm width, with the resulting histogram shown in Figure 3A and the corresponding FEP in Figure 3B.

The simulations provide sufficient sampling to get an almost complete FEP (Δ*G*(*z*)), with the exception of the *z* < 0.55 nm range. The maximum in the probability density function occurs for the [1.5 nm, 1.6 nm] and [1.6 nm, 1.7 nm] bins (Figure 3A), which corresponds to the equilibrium position with Δ*G*(*z*_eq_) = 0 kJ/mol (Figure 3B). At *z* = 2.75 nm, a second well-defined enrichment of probability density is observed. This suggests that the metal complex at the membrane surface is stabilized in comparison to bulk water. The stabilization energy calculated from the FEP is only 1.56 kJ/mol, with no energy barrier for equilibration with bulk water, and this transition is observed very frequently during the simulations. A small energy barrier (2.21 kJ/mol) is however observed on the path from the surface toward insertion of the metal complex in the membrane, at *z* = 2.25 nm. The energy barrier for desorption is also low (about 5 kJ/mol), which explains the high number of insertion/desorption events observed during the simulations (Figures S9 and S10).

From the FEP profile, the partition coefficient (*K*_P_) can be obtained. It can simply be described as the ratio between the concentration of the solute in the membrane ([solute]_mem_) and the concentration of the solute in the aqueous medium ([solute]_w_) at equilibrium. The equilibrium partition coefficient is also correlated with the Gibbs free energy:
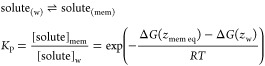
2where Δ*G*(*z*_mem eq_) – Δ*G*(*z*_w_) is the Gibbs free energy for the transfer of the solute from water to the equilibrium position in the membrane. This simple formulation to determine *K*_P_ considers a constant Δ*G*(*z*_mem eq_) for all the membrane *z* positions.^78^ However, this does not account for the nonconstant Gibbs free energy at the different positions in the membrane. For that reason, a more accurate approach to calculate *K*_P_ involves the integration of the free energy profile along the *z* distances from the membrane COM:^79−81^
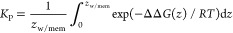
3where *z*_w/mem_ represents the transverse location of the water/membrane interface. ΔΔ*G*(*z*) represents the Gibbs free energy difference for the transfer of the solute from bulk water to a specific location *z* in the membrane and is obtained by
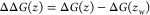
4

Alternatively, *K*_P_ can be calculated from the ratio between the concentrations in the membrane and in water, each of them obtained from the probability density function:
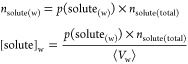
5where *n*_solute(w)_ is the amount of solute in water, *p*(solute_(w)_) is the overall estimated probability of finding the solute in water, *n*_solute(total)_ is the total amount of solute in the system, and ⟨*V*_w_⟩ is the estimated volume of the aqueous medium. Proceeding analogously for the concentration of the solute in the membrane, *K*_P_ can then be obtained by
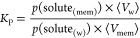
6where *p*(solute_(mem)_) is the estimated overall probability of finding the solute in the membrane and ⟨*V*_mem_⟩ the estimation of the membrane volume. In a MD simulation rectangular box system, the *xy* areas for the water and lipid bilayer slabs are equal, and the previous equation can be further simplified to
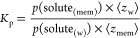
7where ⟨*z*_w_⟩ and ⟨*z*_mem_⟩ are the estimated widths of the water and bilayer slabs in the *z* direction, respectively.

The water/membrane interface was defined as the transverse distance from the membrane COM above which FEP becomes constant, which corresponds to *z* = 4.0 nm (Figure 3B). At larger distances, the solute is not influenced by the presence of the membrane and is therefore considered to be in bulk water. This definition of *z*_w/mem_ aligns with what is expected when *K*_P_ is determined experimentally. Any change in the property under study compared to the same propriety in aqueous solution indicates association of the solute with the lipid membrane.

Both eqs 3 and 7 resulted in *K*_P_ = 1.5, which is unsurprising because they are actually equivalent, as shown in Appendix S.I.3. With eq 2, *K*_P_ = 5.2 was obtained, higher than from the other equations. In spite of the different values obtained when using the distinct approaches, all *K*_P_ calculated led to very low values, but within the same order of magnitude. The exact value of *K*_P_ could not be validated experimentally because to have a significant amount of [Gd(DOTA)]^−^ associated with the membrane, a volume of the membrane phase of at least 10% would be required, which cannot be achieved with liposomes. Attempts to obtain *K*_P_ through the measurement of the heat evolved due to the interaction (using isothermal titration calorimetry) and through effects of [Gd(DOTA)]^−^ on water relaxivity showed no significant variation up to a lipid concentration of 25 mM (*V*_mem_ = 2% of total volume). This indicates that *K*_P_ is lower than 10, in agreement with the estimates obtained from MD simulations.

The FEP shown in Figure 3B shows that membrane-inserted [Gd(DOTA)]^−^ is able to sample part of the membrane hydrophobic core. However, the steep increase in free energy when moving from the equilibrium position toward the bilayer center only allowed correct sampling down to *z* = 0.55 nm, corresponding to an increase in free energy of 20 kJ/mol. This indicates a very large energy barrier for membrane permeation, in agreement with the observed negligible permeability through the intact BBB.^10^

#### 3.2.2 [Gd(DOTA)]^−^ Dynamics

From the previous section and Figures S9 and S10, it was verified that the interaction of the metal complex with the membrane is characterized by fast dynamics of insertion and desorption events and a low partition coefficient to the membrane. Despite that, there are several events where [Gd(DOTA)]^−^ remains inserted in the membrane for a significant amount of time, allowing the calculation of its equilibrium position and orientation when inserted. This allows studying the dynamics of insertion/desorption events, the details of the interaction between [Gd(DOTA)]^−^ and the membrane, and its properties as contrast agent for MRI.

**Figure 4:**
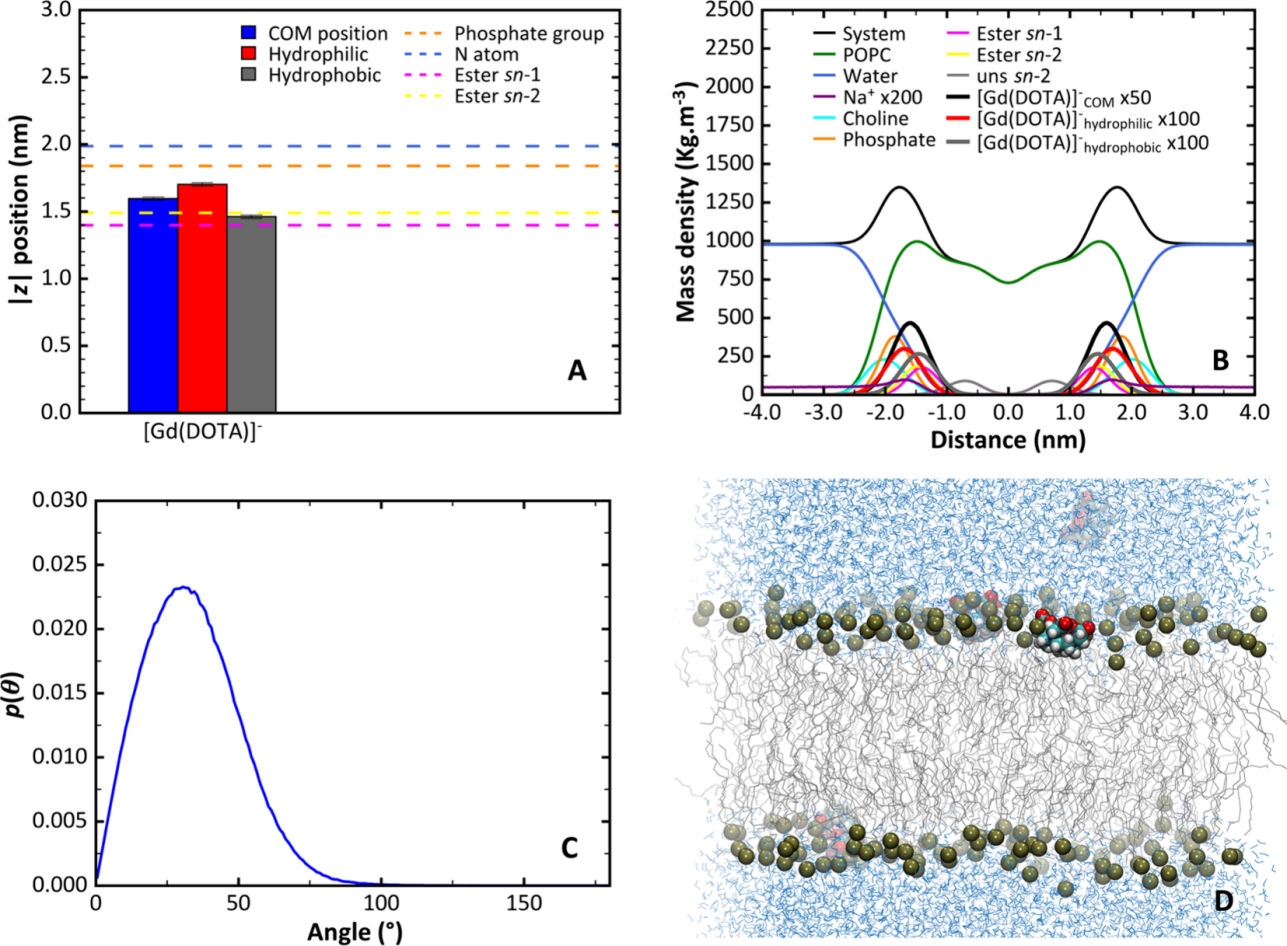
Equilibrium position and orientation of [Gd(DOTA)]^−^ when inserted in the membrane. Equilibrium position of the COM, hydrophilic and hydrophobic portions of the chelate (illustrated in Figure 1C) while inserted in the membrane. The global positions of relevant lipid groups (phosphate, choline, ester *sn*-1, ester *sn*-2 groups) in a POPC bilayer with [Gd(DOTA)]^−^ system (Table 3) are shown in dashed lines (A). Partial mass density profiles along the *z* distance from the membrane COM of the different lipid and [Gd(DOTA)]^−^ groups (B). Probability density function of the angle formed between the vector defined by the hydrophobic and hydrophilic portion of the chelate and the *z* axis (C). Snapshot from one MD simulation with [Gd(DOTA)]^−^ and the P atoms of POPC depicted as van der Waals spheres, the latter with a tan color (D).

The equilibrium position and orientation of the [Gd(DOTA)]^−^ while inserted in the membrane (see section 2 for the definition of an insertion event) was done taking into account the 6 replicates. As can be seen in Figure 4A, the *z* position of the COM of [Gd(DOTA)]^−^ lies on average at 1.60 ± 0.01 nm from the membrane COM, close to the positions of the POPC ester groups. The preferential locations of [Gd(DOTA)]^−^ include the whole headgroup region, down to the initial carbons of the acyl chains (Figures S9 and S10). As expected, the hydrophilic region is on average located at a shallower position than the hydrophobic part of the metal complex. The same conclusion is obtained from the density profile (Figure 4B), where the partial mass density of the chelate extends down to the double bond of the *sn*-2 tail of POPC. The average angle between the vector defined by the hydrophobic and the hydrophilic planes of [Gd(DOTA)]^−^ (illustrated in Figure 1C) and the membrane normal (the *z* axis) is 33.7 ± 0.3° (Figure 4C). As expected, the hydrophobic part of the metal complex is facing the hydrophobic core of the membrane, and the hydrophilic part is facing the aqueous medium, with the amphiphilic moment of [Gd(DOTA)]^−^ aligned with that of the membrane monolayer. A snapshot illustrating the position and orientation of [Gd(DOTA)]^−^ is shown in Figure 4D.

**Figure 5:**
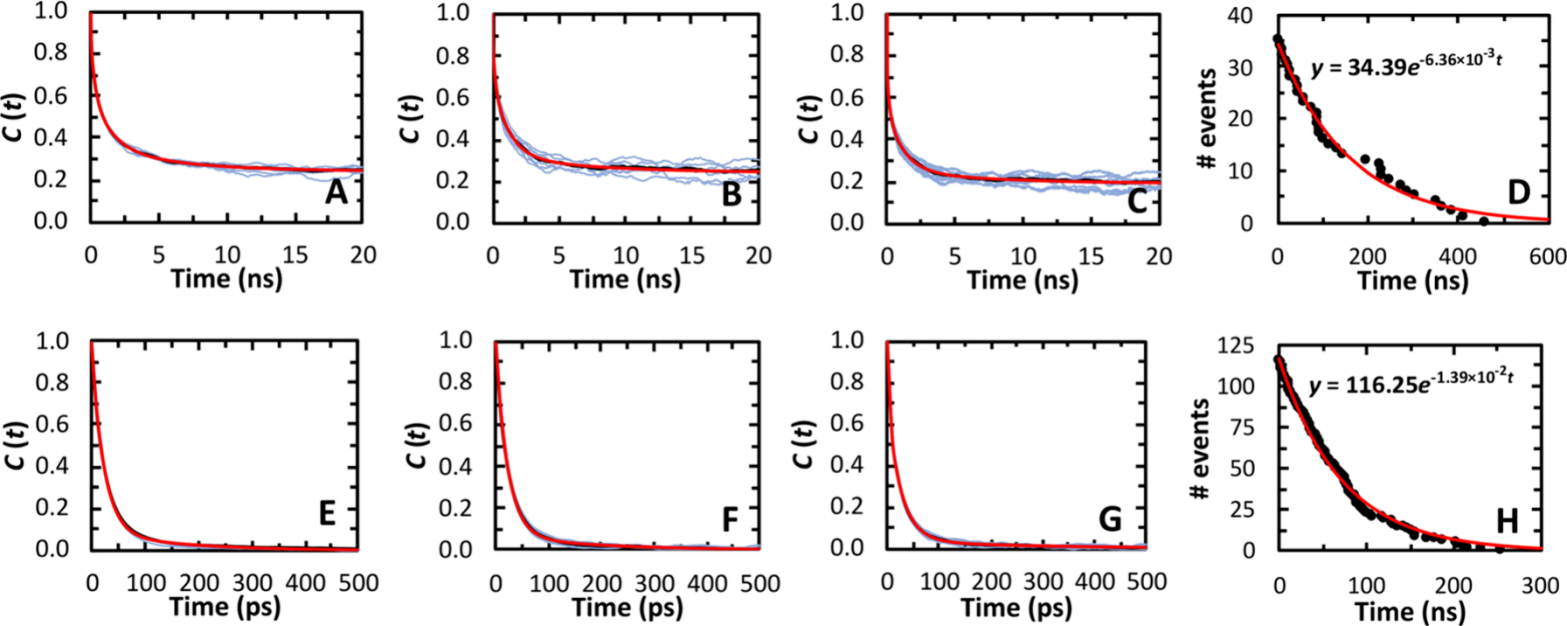
(A–C, E–G) Rotational correlation functions for normal vector of the Gd^3+^-coordinated oxygen atoms plane while [Gd(DOTA)]^−^ is inserted in the membrane (A) or in water (E); the vector Gd–O_water_ for the water directly coordinated with the Gd^3+^ while the metal complex is inserted in the membrane (B) or in water (F); the vector Gd–H_water_ for the water molecule directly coordinated with the Gd^3+^ while the metal complex is inserted in the membrane (C) or in water (G). The blue lines represent *C*(*t*) for each individual metal complex sampling event. The black line represents the weighted average of the individual *C*(*t*) and the red line is the best fitting curve with the parameters of the sum-of-exponentials fitting curve presented in Table S1. (D, H) Residence lifetime of the inner sphere water for the metal complex while inserted in the membrane (D) or in water (H). The black points represent the remaining number of water exchange events observed in the trajectory after one event occurs at that time, starting from the total number of events observed. The red line is the best fitting curve with the equation written in the plot.

As explained in the introduction and Appendix S.I.4, the water proton relaxivity, which governs the efficiency of the Gd^3+^ complex as contrast agent in MRI, is strongly dependent on the rotational correlational time (τ_R_) and the residence time (τ_m_) of the coordinated water in the inner sphere of Gd^3+^. Significant changes were observed in these properties accompanying the transfer of [Gd(DOTA)]^−^ from water to the lipid bilayer (Table 2 and Figure 5).

**Table 2:** Average Rotational Correlational Times (τ_R_; See Table S1 for Further Details) and Residence Lifetime of the Inner-Sphere Water (τ_m_) for [Gd(DOTA)]^−^ in Water or Inserted in the Membrane^a^

[Gd(DOTA)]^−^ location	τ_R_ O_coord_ plane (ps)	τ_R_ Gd–O_water_ vector (ps)	τ_R_ Gd–H_water_ vector (ps)	τ_R_ Gd–H_water_/τ_R_ Gd–O_water_	τ_m_ (ns)
Water	35.6 (1.2)	34.3 (2.1)	28.5 (2.7)	0.83 (0.09)	72.0 (1.7)
Inserted in the membrane	1581 (75)	1440 (122)	1072 (83)	0.74 (0.09)	157 (9.4)

a The rotational correlational times are calculated using second-rank Legendre polynomials of the autocorrelation function. Values between parentheses represent maximal amplitudes of the respective 95% confidence intervals obtained from the fitting.

Similarly to our previous work,^18^ we calculate τ_R_ for the plane defined by the oxygens coordinated to Gd^3+^ (τ_R_ O_coord_ plane), as well as for the vectors between the Gd^3+^ and the oxygen (τ_R_ Gd–O_water_) or hydrogen (τ_R_ Gd–H_water_) of the inner sphere water. The corresponding τ_R_ values obtained while [Gd(DOTA)]^−^ is in water were 35.6, 34.3, and 28.5 ps, respectively. We verify that the τ_R_ values of the plane defined by the coordinated oxygens to the Gd^3+^ are similar to that obtained with the Gd^3+^-O_water_ vector. This similarity was previously observed and interpreted as the inner sphere water being an integral part of the coordination sphere of Gd^3+^, since a strong interaction is established between the oxygen of the inner sphere water and the Gd^3+^ metal ion.^18,82^ A shorter τ_R_ was also obtained for the Gd–H_water_ vector compared to the Gd–O_water_ vector, with a ratio between them of 0.83, as verified previously experimentally by Dunand et al. (with a ratio determined to be 0.65 ± 0.2)^83^ and in our previous simulation work.^18^

While [Gd(DOTA)]^−^ is inserted in the membrane, the τ_R_ obtained for the O_coord_ plane, as well as for the vectors between the Gd^3+^ and the oxygen or hydrogen of the inner sphere water, were 1581, 1440, and 1072 ps, respectively. Thus, when [Gd(DOTA)]^−^ is inserted, its τ_R_ increases from the picosecond to the nanosecond time scale (Table 2 and Figure 5). This is expected, since [Gd(DOTA)]^−^ establishes interactions with the membrane that slow down its rotational motion. A shorter τ_R_ for the Gd–H_water_ vector was observed compared to the Gd–O_water_ vector, with a similar, although slightly lower ratio to that obtained in water. The fitting of the rotational autocorrelation function *C*(*t*) for membrane-inserted [Gd(DOTA)]^−^ reveals a residual term (*a*_∞_) (Figure 5 and the fitting parameters presented in Table S1). This results from the hindered rotational motion of the chelate in the bilayer, normally interpreted as “wobbling”.^78,84^ Another noted feature is the increase of the rotational correlation time of the plane of coordinated oxygen atoms compared to the Gd–O_water_ vector, while in water negligible differences were observed between these two rotational correlation times. This increase is associated with the significant hindrance in the motion of membrane-inserted [Gd(DOTA)]^−^. Since the motion of the inner sphere water molecule is not as severely hindered, the autocorrelation of the vector Gd^3+^–O_water_ decays faster than the corresponding normal vector to the plane of coordinated oxygens of the metal complex while inserted in the membrane.

The values of τ_R_ obtained in this work for [Gd(DOTA)]^−^ in the aqueous media are somewhat larger than previously obtained in the absence of the lipid membrane (τ_R_ for the O_coord_ plane, Gd–O_water_ vector, and Gd–H_water_ vector of 23.7, 23.8, and 19.8 ps, respectively).^18^ This is surprising, given the higher temperature considered in the present study (310.15 K instead of 298.15 K) which was expected to lead to lower correlation times. This unexpected result is due in part to contamination with [Gd(DOTA)]^−^ close to the membrane surface, at |*z*| ≅ 2.75 nm, which equilibrates very fast with [Gd(DOTA)]^−^ in the water. This region corresponds to water involved in lipid solvation, with slower dynamics than bulk water,^85^ which may also contribute to the longer τ_R_ values. Experimentally, τ_R_ can be obtained from the NMRD profile and ^17^O NMR experiments, and 77 ps has been reported at 298.15 K, alongside an activation energy for rotation of 16.1 kJ/mol.^86^ From these values, τ_R_ = 60 ps can be inferred at 310.15 K. This result cannot be directly compared to those obtained here by simulation, since the TIP3P water model used in this work has a self-diffusion coefficient estimated to be two times higher than the experimental one at 310.15 K.^87^ Nevertheless, the experimental and calculated values are in the same order of magnitude. Remarkably, if we apply a 2.0× correction factor to the simulation estimates, based on the self-diffusion coefficient difference, values in the 56–70 ps range are obtained, in close agreement with the experiment.

Concerning the residence lifetime of the inner sphere water (τ_m_), a value of 72 ns was obtained for [Gd(DOTA)]^−^ in water. Taking into consideration the experimental values τ_m_ = 244 ns at 298.15 K and Δ*H*^‡^ = 49.8 kJ/mol,^86^ τ_m_ = 108 ns is estimated at 310.15 K. This value compares very well with the one obtained in this work. On the other hand, when [Gd(DOTA)]^−^ is inserted into the membrane, the residence time of the inner sphere is doubled, to a value of 157.2 ns. This shows that the interaction of the inner sphere water is stabilized by the surrounding lipid groups (details in section 3.2.4).

Taking into consideration the Swift–Connick and SBM theories, the increase of the rotational correlation time, together with the increase of the residence lifetime of the water when the metal complex is inserted in the membrane, are expected to increase the longitudinal relaxation rate of the bound water (1/*T*_1m_) (see Appendix S.I.4). This leads to an increase in the relaxivity induced by the contrast agent on the surrounding water protons. However, at the same time, the increase of the residence lifetime of the inner sphere water will decrease the transmission of the paramagnetic effect to the bulk protons, contributing to a decrease in the relaxivity. Since we are in a fast water exchange regime, in eq 37 of Appendix S.I.4, the *T*_1m_ term (in the microsecond range) will dominate over the residence lifetime of the inner sphere water (τ_m_, in the nanosecond range), and consequently, the overall effect of membrane insertion will be an increase in the relaxivity. This was observed experimentally by Kielar et al. with [Gd(DOTAGA-C_12_)]^−^, a [Gd(DOTA)]^−^ derivative with a linear saturated alkyl chain of 12 carbons. For the bilayer-inserted chelate, these authors obtained a relaxivity (*r*_1_) of 14 mM^–1^ s^–1^ at 310.15 K, measured at 0.47 T (20 MHz),^11^ significantly higher than that of the monomeric state of lipophilic [Gd(DOTA)]^−^ derivatives.^11,88^ Using this value as an approximation, together with the *K*_P_ obtained for [Gd(DOTA)]^−^, we can estimate the overall relaxivity of [Gd(DOTA)]^−^ in a liposome suspension. Assuming additivity of the relaxivities within the water and membrane media, *r*_1_ can be obtained from^89^
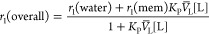
8where *r*_1_(water) is the relaxivity of [Gd(DOTA)]^−^ in water (*r*_1_ = 3.4 ± 0.2 mM^–1^ s^–1^ at 313.15 K at 0.47 T),^90^*r*_1_(mem) is the relaxivity of the metal complex inserted in the membrane, *V̅*_L_ is the molar volume for POPC (0.765 M^–1^) and [L] is the concentration of POPC. The overall relaxivity estimated for the [Gd(DOTA)]^−^ at 10 mM lipid would increase from 3.4 mM^–1^ s^–1^ in water to 3.5 mM^–1^ s^–1^ in the presence of liposomes. This small increase is within the experimental uncertainty and could not be observed experimentally (data not shown). However, it should be noted that very high lipid concentrations may be found *in vivo* in some tissues, potentially leading to increased association of [Gd(DOTA)]^−^ with membranes and thus increased relaxivity.

#### 3.2.3 Effect of [Gd(DOTA)]^−^ on Membrane Properties

**Figure 6:**
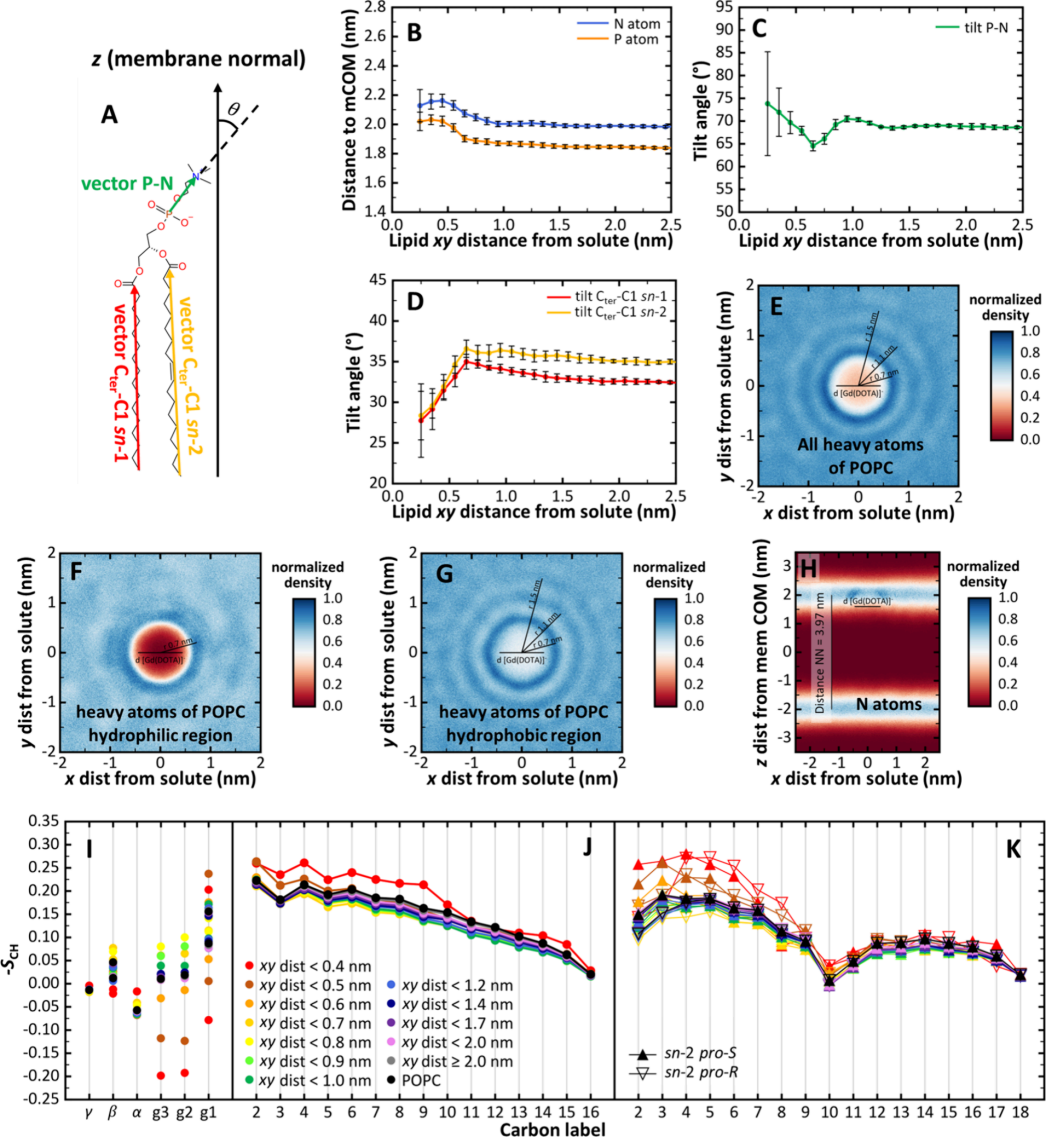
Local membrane properties for varying *xy* distance of POPC from [Gd(DOTA)]^−^. Schematic representations of vectors used to define the tilt angles θ with the lipid bilayer normal (A). Distance of the P and N atoms of POPC to the membrane COM for varying *xy* distance with bins of 0.1 nm (B). P–N tilt angle of POPC (C) and tilt angle of the vector C_ter_-C1 of the *sn*-1 and *sn*-2 chains of the POPC molecules (D), relative to the lipid bilayer normal. Normalized density maps for all heavy atoms of POPC (E), all heavy atoms of the POPC headgroup (including the ester groups of the acyl chain) (F) and all heavy atoms of the hydrophobic acyl chains (from C2 to C_ter_) (G) in the same monolayer where the [Gd(DOTA)]^−^ is located, as a function of the *x* and *y* distances from the [Gd(DOTA)]^−^ COM. These previous density maps are averaged over the *z* axis. Normalized density maps for the N atom of POPC for varying *x* distance from [Gd(DOTA)]^−^ and *z* distance from the membrane COM averaged over the *y* axis (H). The latter density map was rotated in order for the lower monolayer to be represented in the upper part of the figure, for better visualization. The line that represents the diameter of [Gd(DOTA)]^−^ corresponds to its actual position in the density maps. The absolute density maps are presented in Figure S16, that also includes the density map of the P atom of POPC in the same way as for the N atom, in both absolute and normalized scales. Order parameters -*S*_CH_ for the POPC headgroup (I), *sn*-1 (J), and *sn*-2 (K) chain calculated for several *xy* distance bins from [Gd(DOTA)]^−^. All of these lateral distances were calculated between the COM of [Gd(DOTA)]^−^ and the phosphorus atom of the POPC molecule.

We now turn to the study of the lipid properties and how they change upon insertion of [Gd(DOTA)]^−^. To obtain a complete picture, both global and local effects were considered, according to the *xy* distance from the solute. Starting with the global properties, the average area per lipid (area of the simulation box divided by the number of lipid molecules) increases in the presence of [Gd(DOTA)]^−^ (Table 3). This is due to the expansion of the simulation box caused by the insertion of [Gd(DOTA)]^−^ in the membrane. To evaluate the effect on the lipids themselves, the contribution from [Gd(DOTA)]^−^ must be removed. It was assumed that one chelate occupies a circular area in the *xy* monolayer plane corresponding to the diameter obtained from the distance between opposite noncoordinate oxygen atoms in the X-ray crystallographic structure,^91^ corresponding to 0.871 nm. We also take into consideration that on average at least one complex is inserted in each monolayer at a given time (Figures S9 and S10). Removing the area of the inserted metal complex leads to an area per lipid of 0.657 nm^2^, which is exactly the value obtained for the pure POPC membrane. The thickness of the bilayer shows a slight decrease in the presence of [Gd(DOTA)]^−^ of 0.017 nm in both *D*_HH_ and *D*_P–P_ and 0.015 nm in *D*_N–N_ (Table 3). The combination of the increase in the area of the membrane and the decrease in thickness helps to accommodate the perturbation caused by the presence of [Gd(DOTA)]^−^ in the lipid bilayer, with the rearrangement of the lipid tails to prevent the potential void beneath the metal complex. Chelate insertion leads to no significant changes on the probability density functions and average tilt angles of the vectors formed between the phosphorus and nitrogen atoms with the lipid bilayer normal (68.7° for the pure POPC system, Figure S11 and Table 3). Slight increases of the average tilts of the POPC *sn*-1 and *sn*-2 C_ter_-C1 vectors relative to the membrane normal (see Figure 6A for the definition of the tilt angles and Figure S12 for their probability density functions) are observed in the system with [Gd(DOTA)]^−^. This suggests a global disordering of the acyl chains in the system with [Gd(DOTA)]^−^. However, chelate insertion leads to no significant changes in proton order parameters (Figure S13). Finally, a slight increase in the calculated volume per lipid is observed for the system with [Gd(DOTA)]^−^, compared to pure POPC, reflecting the volume occupied by the chelate in the membrane. The overall increases in area and volume per lipid indicate a slightly expanded bilayer and a concomitant looser packing of lipid molecules.

**Table 3:** Global Properties of the Lipid Bilayer in the Presence and Absence of [Gd(DOTA)]^−^ Complexes at 310.15 K^a^

	area/lipid (nm^2^)	*D*_HH_ (nm)	*D*_P–P_ (nm)	*D*_N–N_ (nm)	*V*/lipid (nm^3^)^b^	P–N tilt angle	*sn*-1 C_ter_-C1 tilt angle	*sn*-2 C_ter_-C1 tilt angle
Pure POPC	0.657 (0.001)	3.695 (0.006)	3.701 (0.006)	3.989 (0.006)	1.310	68.7°	31.9°	34.6°
POPC + 4 [Gd(DOTA)]^**–**^	0.663 (0.002)	3.678 (0.008)	3.684 (0.008)	3.974 (0.008)	1.317	68.6°	32.5°	34.9°

a Maximal amplitudes of the respective 95% confidence intervals are given in parentheses.

b The volume occupied per lipid molecule is calculated by multiplying the area/lipid by half the *D*_N–N_.

The global properties of the lipid bilayer can provide some clues on the effects caused by the insertion of solutes in the membrane. However, they do not convey the full picture of what is happening locally with the lipid molecules close to the membrane-inserted solute. For that reason, we investigated several properties of the lipid molecules as a function of the lateral distance from the [Gd(DOTA)]^−^, calculated as the *xy* distance between the COM of the chelate and the phosphorus atom of POPC.

First, we looked at the distance of the nitrogen (*D*_N-mCOM_) and phosphorus (*D*_P-mCOM_) atoms of POPC to the COM of the membrane (Figure 6B). For neighboring POPC molecules (*xy* < 0.6 nm), higher average *D*_N-mCOM_ and *D*_P-mCOM_ distances are observed. This corresponds to a local increase in membrane thickness (*D*_P-mCOM_ = 2.02 nm and *D*_N-mCOM_ = 2.16 nm at a *xy* distance of 0.45 nm), to values even higher than in pure POPC (*D*_P-mCOM_ = 1.85 nm and *D*_N-mCOM_ = 1.99 nm, Table 3). For longer *xy* distances, *D*_N-mCOM_ and *D*_P-mCOM_ decrease and reach plateau values, corresponding to the global ones for the system with [Gd(DOTA)]^−^ (Table 3). It should be noted that the nearest neighbors of [Gd(DOTA)]^−^ are strongly outnumbered by those at longer distances, and hence global averages are dominated by the asymptotic values. With regard to the P–N tilt angle of POPC (Figure 6C), larger values are observed at close proximity to [Gd(DOTA)]^−^ (*xy* = 0.45 nm) compared to the pure POPC system (67.8°), indicating vector orientations closer to the bilayer plane with reduced vertical difference between the N and P atoms, compared to the value at long distances (Figure S14). This feature, combined with the increase of the local thickness, suggests an “engulfment” of [Gd(DOTA)]^−^ by the lipid headgroup, consistent with a possible interaction between [Gd(DOTA)]^−^ and the choline group, as addressed in the next section. The value of the P–N tilt angle decreases to 64.5°, lower than observed for the pure POPC system, at a lateral distance of 0.65 nm from [Gd(DOTA)]^−^. This low tilt angle indicates an increased alignment of the P–N vector with the bilayer normal, suggesting a more extended conformation between the phosphate and choline group. For 0.65 nm < *xy* < 0.95 nm, the tilt angle increases to ≅ 70°, again higher than for pure POPC. These variations suggest that the perturbation induced by [Gd(DOTA)]^−^ at very short distances is overcompensated in subsequent lipid layers, generating a spatially “oscillatory” pattern which is gradually attenuated and vanishes asymptotically. This behavior is also apparent in the probability density functions of the P–N tilt angle, which undergo slight displacements for different distance ranges (Figure S15A).

Looking now at the density map calculated for all POPC heavy atoms (Figure 6E), a significant reduction of relative density is observed in the location where the chelate is inserted (of 56% relative to the value at an absolute distance of 2.0 nm). The reduction is most accentuated in the headgroup region of the phospholipids (Figure 6F), compared to the acyl chains (Figure 6G), which are decreased by 97% and 29%, respectively. These results clearly indicate that [Gd(DOTA)]^−^ creates a void in the hydrophilic region, with a small reduction in the hydrophobic core of the membrane. This is a consequence of the relative position of the chelate COM in the lipid bilayer, slightly above the ester groups (Figure 4A). The small decrease in the hydrophobic region is explained by the vertical fluctuation of [Gd(DOTA)]^−^, with its mass density being observed down to the location of the unsaturated bond of the *sn*-2 chain (Figures 4B, S9, and S10). Another feature observed in the density map of the hydrophilic region of POPC is the significant increase at a lateral distance of 0.7 nm. This is especially notable for the N atoms (Figure 6H), which display a well-defined enrichment around the location of [Gd(DOTA)]^−^. The increase occurs at distances *z* from the membrane COM larger than the global *D*_N–N_, in accordance with the previously described local thickness increase. For the phosphorus atom, the corresponding density increase is more diffuse (Figure S16E, F), suggesting that interaction between [Gd(DOTA)]^−^ and lipid head groups occurs through the choline group.

The proton order parameters (-*S*_CH_) of the lipid headgroup for varying lateral distance from the chelate allow a fine characterization of the local properties in this lipid bilayer region (Figure 6I). At close proximity (*xy* < 0.6 nm), the order parameters of the glycerol carbons are strongly negative, clearly lower than the corresponding values for pure POPC. Therefore, the C–H bond vector of these carbons aligns with the lipid bilayer normal, and the glycerol moiety is mostly parallel to the membrane plane. The lower |*S*_CH_| of the choline α and β carbons is in line with the previously described high P–N tilt angle and consequent engulfment of [Gd(DOTA)]^−^ by the choline group. The bending of the lipid headgroup toward the chelate is also facilitated by the conformation adopted by glycerol carbons. A close inspection of the headgroup -*S*_CH_ variation reveals the same spatially oscillatory pattern previously described for other properties (Figure 6B, C, E, and G).

We now turn to the local properties of the hydrophobic acyl chains, starting from the tilt angle of the *sn*-1 and *sn*-2 C_ter_-C1 vectors relative to the lipid bilayer normal (Figure 6D). Close to the chelate (*xy* < 0.45 nm), the tilt angles of both chains (27.7° and 28.4° for the *sn*-1 and *sn*-2 chain at 0.25 nm, respectively) are significantly lower than in pure POPC (Table 3). This indicates extended conformations of both acyl chains, leading to an increase in the local ordering (Figures 6J, K). The tilt angles increase until their maximal values of 35.0° and 36.6° for the *sn*-1 and *sn*-2 chain, respectively, at *xy* = 0.65 nm. These values are higher than those obtained for pure POPC, pointing to a less extended conformation in both acyl chains and a consequent local disordering. For longer *xy* distances, the tilt angles of both chains decrease asymptotically to the global values. Concomitant variations can be observed in the acyl chain -*S*_CH_ profiles. At close range, both chains have an increase in the order parameters to similar values in the first segments. A slight increase in order parameter of the unsaturated bond of the chain is also evident, especially at the carbon C10. Overall, this confirms that [Gd(DOTA)]^−^ induces extended acyl chain conformations, with consequent increase in local ordering close to the solute. This allows a tighter packing and suggests specific interactions between the lipid acyl groups and [Gd(DOTA)]^−^. At a *xy* distance between 0.6 and 0.7 nm, where the highest tilt angles of the acyl chains were registered, minimal values of -*S*_CH_ are observed for both acyl chains, again reflecting the spatially oscillatory pattern around the chelate.

**Figure 7:**
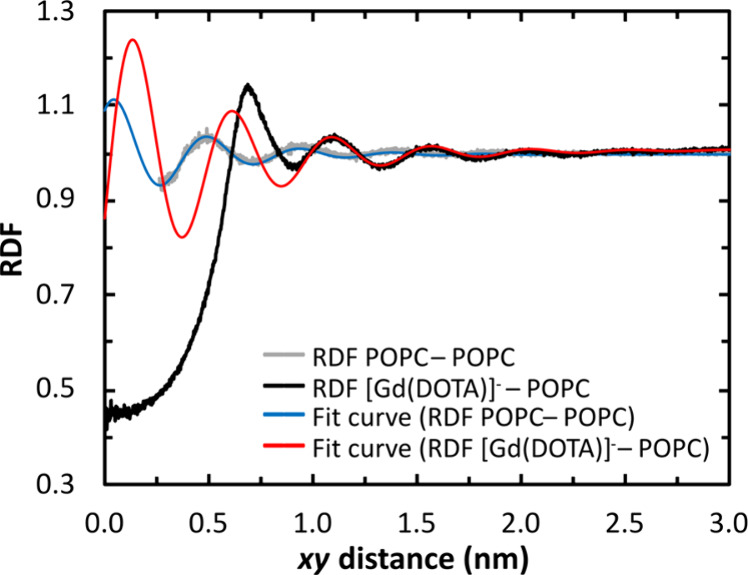
RDFs in the *xy*-plane of all POPC heavy atoms in the same monolayer where the solute is located around the COM of [Gd(DOTA)]^−^ (black line) and of all POPC heavy atoms of the lipid bilayer around each heavy atom of POPC (gray line). In the latter RDF profile, data below 0.26 nm are not shown since they correspond to intramolecular particle distances. Each RDF is fitted with a sinusoidal damped function (red and blue lines), with the fitting parameters in Table S2. The positions of the most relevant peaks in these RDF profiles are detailed in Table S3.

Albeit in the fluid state, the membrane is a medium with local structure and this justifies in part the spatially oscillatory pattern observed. In Figure 7, the RDF calculated for all POPC heavy atoms around each other in a pure lipid membrane is compared with that around [Gd(DOTA)]^−^ in a membrane containing chelates. In the case of pure POPC, the RDF is well described by a damped sinusoidal function (Figure 7 and Table S2), with a characteristic length (2π/ω, where ω is the recovered frequency) of 0.446 nm, very close to experimental determinations of the lateral spacing in fluid phosphatidylcholine bilayers obtained from wide-angle X-ray scattering (around 0.45 nm).^92−94^ In the case of the POPC RDF around the COM of [Gd(DOTA)]^−^, a clear misfit is obtained for *xy* distances lower than 0.95 nm, with a depletion at *xy* < 0.6 nm and an enrichment for 0.6 nm < *xy* < 0.95 nm. The depletion region is a trivial consequence of the presence of the chelate that occupies the hydrophilic region of the membrane. The higher amplitude and lower width of the first peak in the RDF of POPC around the chelate (*xy* ≈ 0.7 nm) reflects the increase in the order of the first layer of POPC molecules. The oscillatory pattern of additional layers of POPC is well described by the sinusoidal damped function, although the parameters are somewhat different from those observed in pure POPC. The characteristic length period is longer (0.478 nm), the oscillation persists for longer distances (*k*_a_ equal to 2.00 nm^–1^ instead of 2.55 nm^–1^ for pure POPC), and a logistic function was required to describe the small increase in the average POPC density as the distance from [Gd(DOTA)]^−^ increases. The longer characteristic length agrees with the disordering observed for POPC located at intermediate distances from the chelate.

#### 3.2.4 Interaction of [Gd(DOTA)]^−^ with Lipid and Water Molecules

In sections 3.2.1 to 3.2.3, it was shown that in spite of the low lipophilicity of [Gd(DOTA)]^−^, the interactions established with the membrane are sufficiently strong to orient the metal complex with its amphiphilic moment aligned with that of the lipid molecules in the same monolayer, and to maintain it inserted in the membrane for relatively long time intervals. It was also shown that although the overall properties of the membrane are not strongly affected by the presence of [Gd(DOTA)]^−^, a local ordering of the POPC molecules is observed. To understand those effects, the interactions established between [Gd(DOTA)]^−^ and the lipids will be analyzed in detail.

**Figure 8:**
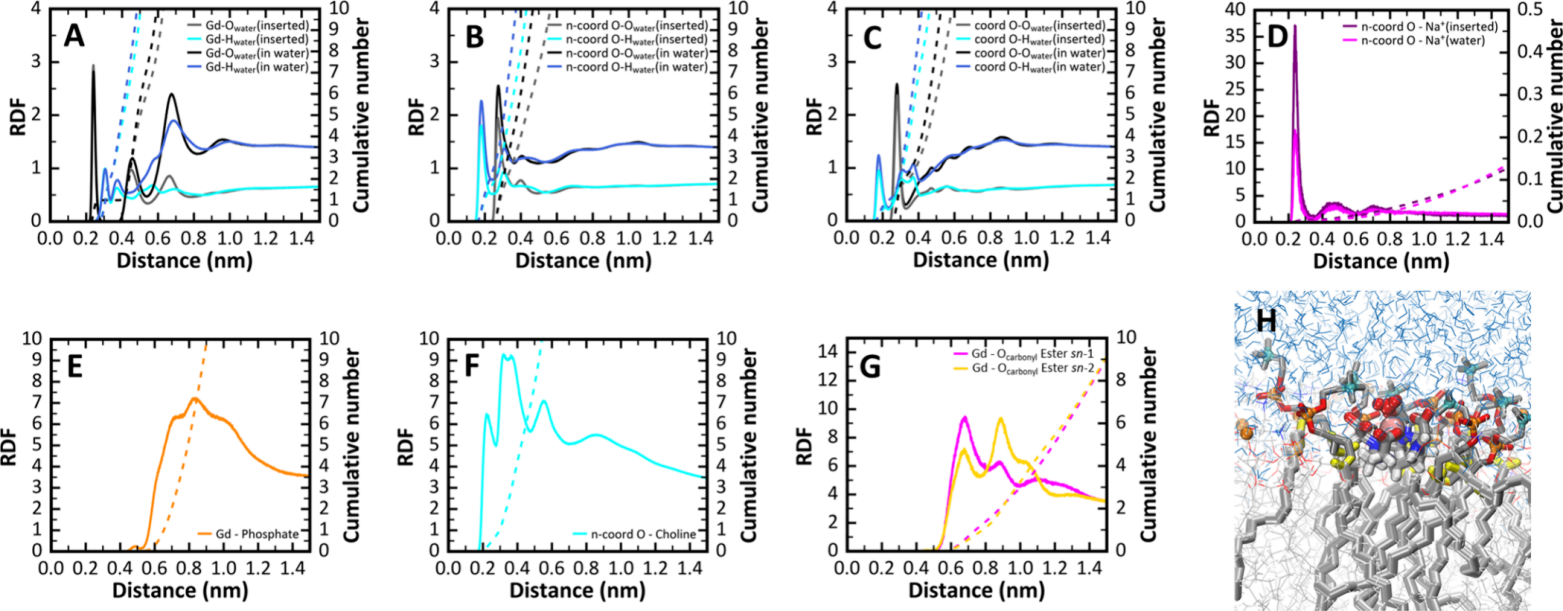
Atom-atom RDF profiles (solid line) and their respective integration to obtain the cumulative number (dashed line with the same respective color) of different groups of the aqueous (A–D) or membrane (E–G) media around [Gd(DOTA)]^−^. In plots (A–D), the profiles were obtained separately while the metal complex is inserted or not into the membrane. In plots (E–G) solely the former situation was considered, only for the monolayer in which the metal complex is inserted. Gd^3+^-O_water_ and Gd^3+^-H_water_ RDF (A), RDFs of water O or H atoms around the non-Gd^3+^-coordinated ligand O atoms (B), water O or H atoms around the Gd^3+^-coordinated ligand O atom (C), and Na^+^ around the non-Gd^3+^-coordinated O atom (D). RDF profile of phosphate group of POPC around the Gd^3+^ metal ion (E), choline group of POPC around the non-Gd^3+^-coordinated ligand O atoms (F), and the carbonyl ester oxygen of the *sn*-1 and *sn*-2 chains of POPC around the Gd^3+^ (G). The positions of the most relevant peaks of all these RDF profiles are detailed in Table S3. Snapshot of a close-up view of the chelate inserted into the membrane, interacting with surrounding lipid groups (an expanded version of this snapshot with higher resolution is presented in Figure S17). Choline (nitrogen atom in cyan), phosphate (the phosphorus atom in orange and the neighboring oxygens in red), and ester groups (yellow) are displayed, as well as the inner sphere water (molecule in red) (H).

**Figure 9:**
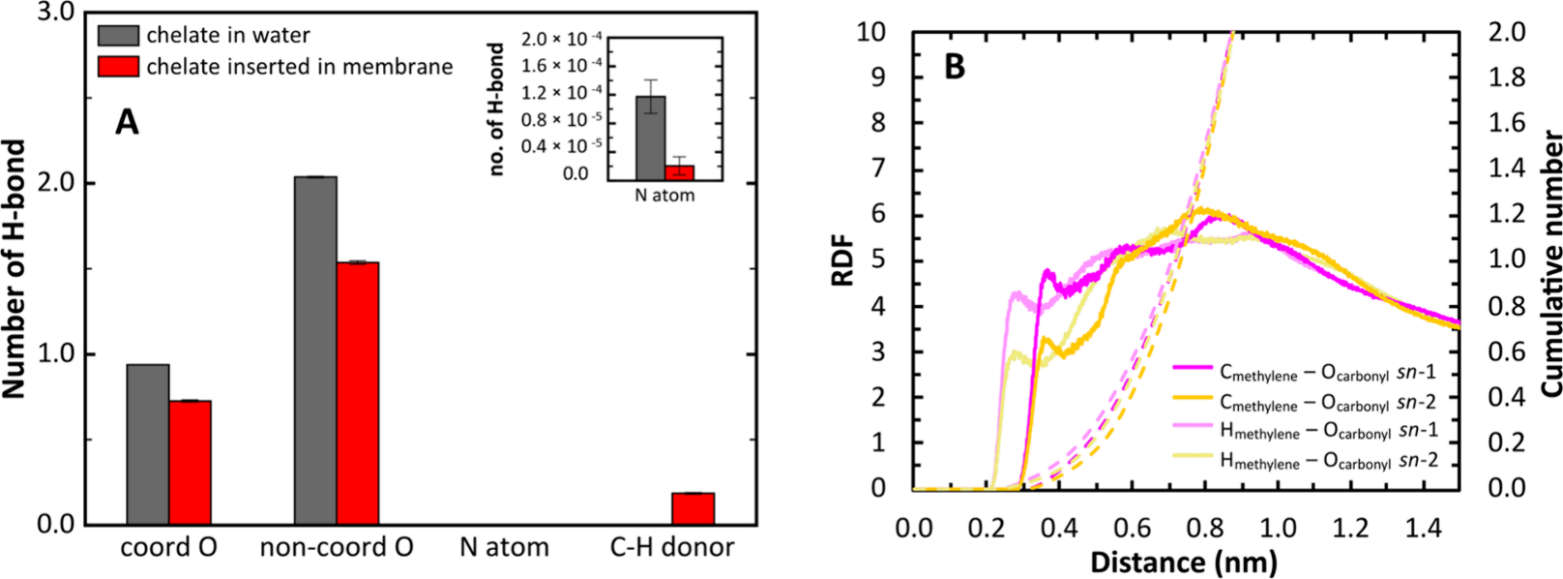
Average number of instant H-bonds per particle of [Gd(DOTA)]^−^ with water molecules, while inserted in the membrane or in the aqueous medium. H-bonds occur with the acceptor atoms of the chelate, namely the Gd^3+^-coordinated and non-Gd^3+^-coordinated O atoms and the N atoms of the tetraaza macrocyclic ring. Average instant number of improper H-bond between all methylene groups (C–H) of the [Gd(DOTA)]^−^ as donors and all oxygen atoms of the POPC ester groups (A). Atom-atom RDF profiles (solid line) with their respective integration to obtain the cumulative number (dashed line with the same respective color) of the carbonyl ester oxygen of the *sn*-1 and *sn*-2 acyl chains of POPC around the methylene carbon and hydrogen atoms of [Gd(DOTA)]^−^ (B). The positions of the most relevant peaks in these RDF profiles are detailed in Table S3.

The RDF profiles of water around water-located [Gd(DOTA)]^−^ are similar to those published in our previous work,^18^ in the absence of lipid bilayer (Figure 8A–C). While the location of the different peaks is identical, there are differences in the shapes of the RDFs, stemming from the anisotropic topology of the membrane-containing system simulated here. For the membrane-inserted chelates, the corresponding RDFs show the expected decrease in the number of water molecules around the solute. The RDF profiles of the water oxygens around Gd^3+^ show that one water molecule is always coordinated in the inner sphere and that the distance is the same when [Gd(DOTA)]^−^ is in the water or inserted in the membrane (Figure 8A). A slight increase in the height of the RDF peak for the coordinated water molecule is observed in the case of membrane-inserted [Gd(DOTA)]^−^. This suggests a stronger interaction and is in agreement with the higher residence lifetime of the coordinated water molecule when [Gd(DOTA)]^−^ is inserted in the membrane (see section 3.2.2). As expected, the coordinated water molecule is always interacting with Gd^3+^ through the oxygen atom, with the density peaks from the corresponding H atoms being at higher distances from Gd^3+^. The distance and orientation of the water molecules in the first and outer hydration layer are also essentially independent of whether [Gd(DOTA)]^−^ is in water or inserted in the membrane. Looking now at the RDF profiles of O and H atoms of water around the ligand Gd^3+^-coordinated and non-Gd^3+^-coordinated oxygens (Figure 8B, C), H atoms show closer proximity, compared to the O atoms of the same water molecules. These first peaks correspond to the occurrence of H-bonds. As seen in Figure 9A, H-bonds are formed between water molecules and both Gd^3+^-coordinated and non-Gd^3+^-coordinated oxygens. Virtually no H-bonds are formed between the N atoms of the [Gd(DOTA)]^−^ and water molecules, especially when the complex is inserted in the membrane. The number of H-bonds formed between the chelate and water molecules is identical to that observed in the previous work when the complex is in water.^18^ For the membrane-inserted chelate, despite the orientation of its hydrophilic part toward the aqueous medium, there is a decrease in the number of H-bonds established with water. This suggests that the membrane is shielding these chelate H-bonding acceptor atoms from water molecules. On the other hand, the RDFs of Na^+^ around the noncoordinated oxygen atoms of [Gd(DOTA)]^−^ show a well-defined peak in both situations, indicating a specific interaction (Figure 8D). Notably, there is a higher density of sodium ions around membrane-inserted [Gd(DOTA)]^−^. This effect is due to the strong interaction of Na^+^ with phospholipid head groups of POPC, leading to higher local density and more likely interaction when the chelate is inserted into the membrane. It should be noted that the common force fields overestimate the interaction between sodium ions and lipids, compared to experimental observations.^95^

When the metal complex is inserted into the membrane, interactions with lipid groups are expected. The RDF profile of the negatively charged phosphate groups around Gd^3+^ does not show any kind of organization suggestive of specific interactions (Figure 8E). However, the RDF of the choline groups around the noncoordinated oxygen atoms of [Gd(DOTA)]^−^ shows well-defined and sharp peaks, the first of which at a distance <0.25 nm, indicating significant interaction between the groups (Figure 8F). An electrostatic interaction between the noncoordinated oxygens of the metal complex and the choline groups is expected since the former have a significant negative partial charge of −0.62*e* (see Table S8 from ref (18)) whereas the latter have a significant overall positive charge of +0.75*e*, according to the force fields used.^31,32^ With regard to the RDF profiles of the carbonyl oxygen of the *sn*-1 and *sn*-2 chains of POPC around Gd^3+^ (Figure 8G), two peaks in each RDF are observed. The first peak appears at 0.6–0.8 nm from the Gd^3+^ with higher intensity for the *sn*-1 chain compared to the *sn*-2 chain. This suggests interactions between the lipid carbonyl groups and the macrocyclic ring. Looking at the methylene groups of [Gd(DOTA)]^−^, a fair polarization is observed in the C–H bonds due to the neighborhood electronegative nitrogen of the amine that transfers charge to the Gd^3+^ metal ion (see Table S8 from ref (18)). For that reason, we calculate the RDFs profiles of the carbonyl oxygen atoms of the *sn*-1 and *sn*-2 chains around all the carbon and hydrogen atoms of the [Gd(DOTA)]^−^ methylene groups (Figure 9B). At close distances, all RDFs present a fairly defined peak, suggesting an interaction between the methylene groups and the POPC ester groups. The carbonyl oxygens of the acyl chains appear at closer distances to the hydrogen than to the carbon atoms of the methylene groups. The distance at which all these peaks appear is compatible with a possible improper weak H-bond with the C–H bond as a donor and the POPC ester oxygens as acceptors. Although these weak interactions are nonconventional, they have been well established for over two decades, with their identification in crystal structures. However, their identification in aqueous solution remains elusive.^96−100^ Nevertheless, several ab initio calculations have shown that the binding energies of these improper H-bonds could vary between 2 and 20 kJ/mol in gas phase, potentially matching the energy of a typical H-bond between water molecules (20 kJ/mol).^97,99,100^ However, these studies also show that the increase of the dielectric constant (ε) rapidly decreases these binding energies, becoming disfavored for ε ≥ 4.3.^100^ However, low dielectric environments can be found, for example, inside a protein (ε ≈ 4^100^) or inside a lipid membrane (ε = 4–20 in the polar region of the membrane and ε = 1–2 in the hydrophobic core),^101^ potentially making these improper H-bonds relevant. Since the transverse equilibrium position of [Gd(DOTA)]^−^ lies between the interface of the hydrophilic and hydrophobic region of the membrane (Figure 4A), we can assume that these methylene groups reside in a low dielectric environment. By employing the same geometrical and orientational criteria for a typical H-bond [distance between donor (D) and acceptor (A) lower or equal than 0.35 nm and ∠HDA ≤ 30°; these are the default criteria in GROMACS],^34−36^ we were able to find a total average of 0.2 of these improper H-bonds at a given time, considering all the [Gd(DOTA)]^−^ methylene groups (Figure 9A). This relatively low number seemingly implies that these improper H-bonds may contribute somewhat to the stabilization of [Gd(DOTA)]^−^ in the lipid bilayer, but the electrostatic interactions appear to be the predominant forces. These interactions between [Gd(DOTA)]^−^ and the ester groups of POPC help explain the higher local thickness of the membrane and the considerable increase in the local ordering (see section 3.2.3).

All the features discussed above can be visualized in the snapshot of Figure 8H. [Gd(DOTA)]^−^ clearly keeps its inner sphere water coordinated. The choline groups are oriented toward the hydrophilic part of the chelate, while the phosphate groups are further away. The snapshot also clearly illustrates [Gd(DOTA)]^−^ sitting on top of the ester groups of neighboring POPC molecules, which display extended acyl chain conformations.

## 4 Conclusions

From this work, several important clues allowed us to understand the interactions between [Gd(DOTA)]^−^ and lipid bilayers. From the highly hydrophilic character of this complex (log *D*_oct/PBS_ = −4.16),^8^ no interaction would be expected. This work demonstrates that the chelate is able to insert into the membrane. Although its partition coefficient to the lipid bilayer is low, it is still 4 orders of magnitude higher than *D*_oct/PBS_, indicating significant chelate-membrane interactions. This shows that it is inappropriate to rely solely on octanol/water partitions for the prediction of solute lipophilicity in drug design. Lipid membranes are complex and anisotropic, and their components establish intricate interactions with each other and with solutes that cannot be described by homogeneous media such as octanol.^102−105^

The preferential transverse location of [Gd(DOTA)]^−^ in the membrane is close to the ester groups of the phospholipid, spanning the region between the phosphate and the initial carbons of the hydrophobic phospholipid chains. The amphiphilic moment of [Gd(DOTA)]^−^ is oriented parallel to that of the lipid monolayer, with the hydrophilic region facing the aqueous medium, thus facilitating the coordination of one inner-sphere water molecule and its exchange with bulk water. This ensures that [Gd(DOTA)]^−^ still works as an efficient MRI contrast agent when associated with the membrane, albeit with changed physical-chemical properties. The rotation correlation times (τ_R_) of membrane-inserted [Gd(DOTA)]^−^ increase over 1 order of magnitude compared to the value in bulk water. Although this could lead to a dramatic increase in water proton relaxivity, the overall effect in membrane suspensions would be small, on account of the very low *K*_P_. Nevertheless, this work shows the possibility of predicting and understanding the changes in these properties relevant to the proton relaxivity upon the partition of Gd^3+^-based complexes into lipid membranes. Such understanding is fundamental in the development, for example, of imaging probes or theranostic agents based on liposome formulations.^6,11−13^

While inserted, the solute induces changes in the local properties of the lipid bilayer. [Gd-DOTA]^−^ is engulfed through the establishment of interactions with the choline and ester groups, causing local (<0.5 nm lateral distance) increases in the P–N tilt angle, bilayer thickness, and acyl chain order parameters. At intermediate lateral distances (0.5–0.8 nm), these effects are reversed. For longer distances, the properties of the lipid bilayer oscillate and asymptotically relax to bulk values, indicative of slight overall disordering, compared to the pure membrane system.

Large efforts have been placed in the development of targeted MRI contrast agents. However, the permeation of these metal complexes through biomembranes is still a main difficulty to overcome.^6^ With this work, we hope to establish a frame of reference for future works involving the interaction with lipid bilayers of metal imaging probes. Such works have the potential to improve the understanding of key aspects in this interaction. This will ultimately be important in the improvement of passive permeability of these compounds through membranes toward the target tissues. Of major relevance is the case of tissues protected by tight endothelia, such as the BBB. Overcoming this problem has unlimited potential in the development of new strategies for the early diagnosis of neurodegenerative diseases, enabling the development of new therapeutic strategies.

## Data Availability

All the necessary files to run the simulations performed in this work are also available in the Zenodo repository, together with a full trajectory (https://doi.org/10.5281/zenodo.11179585); other full trajectories are available upon request.

## Supporting Information Available

Block analysis method for error estimation in correlated data (Appendix S.I.1), error estimation for a weighted mean (Appendix S.I.2), partition coefficient calculation (Appendix S.I.3), and the relaxivity theory (Appendix S.I.4) and additional figures and tables, with the results of complementary simulation analyses (PDF)
All the necessary files to run the simulations performed in this work: Initial (after a minimization step to remove any bad contact) and final structures (*.gro), GROMACS topology files (*.top, *.itp), and GROMACS molecular dynamics parameters files (*.mdp), accompanied by a text file description (readme.txt) (ZIP)
