# Supplementary material for: Interaction of MRI Contrast Agent [Gd(DOTA)]− with Lipid Membranes: A Molecular Dynamics Study
Source: Inorg Chem. 2024 May 25;63(24):10897–914. doi: 10.1021/acs.inorgchem.4c00972 (PMC11186012; doi:10.1021/acs.inorgchem.4c00972)
Supplement: Supplementary file 1 — ic4c00972_si_001.pdf [file ic4c00972_si_001.pdf]

Supporting Information for:

Interaction of MRI contrast agent $[Gd(DOTA)]^-$ with lipid membranes: a molecular dynamics study

Alexandre C. Oliveira[a,b*], Hugo A. L. Filipe[a,c], Carlos F.G.C. Geraldes[a,d,e], Gregory A. Voth[f], Maria João Moreno[a,b,h*], Luis M. S. Loura[a,g,h*]

[a] Coimbra Chemistry Centre, Institute of Molecular Sciences (CQC-IMS), 3004-535 Coimbra, Portugal

[b] Department of Chemistry, University of Coimbra, 3004-535 Coimbra, Portugal

[c] CPIRN-IPG—Center of Potential and Innovation of Natural Resources, Polytechnic Institute of Guarda, 6300-559 Guarda, Portugal

[d] Department of Life Sciences, University of Coimbra, Calçada Martim de Freitas, 3000-393 Coimbra, Portugal

[e] CIBIT/ICNAS - Instituto de Ciências Nucleares Aplicadas à Saúde, Pólo das Ciências da Saúde, Azinhaga de Santa Comba, 3000-548 Coimbra, Portugal

[f] Department of Chemistry, Chicago Center for Theoretical Chemistry, James Franck Institute, and Institute for Biophysical Dynamics, University of Chicago, Chicago, Illinois 60637, United States

[g] Faculty of Pharmacy, University of Coimbra, 3000-548 Coimbra, Portugal

[h] CNC—Center for Neuroscience and Cell Biology, University of Coimbra, 3004-517 Coimbra, Portugal

Keywords: Contrast Agents for MRI; Molecular Dynamics Simulation; Lipid bilayers; Partition; Water proton relaxivity

Table of contents

Contents **Page numbers**

Contents	Page numbers
Appendix S.I.1 Block analysis method for error estimation in correlated data	S2
Appendix S.I.2 Error estimation for a weighted mean	S5
Appendix S.I.3 Partition coefficient calculation	S6
Appendix S.I.4 Relaxivity theory	S9
Additional Figures and Tables	S13
References	S27

Appendix S.I.1 Block analysis method for error estimation in correlated data

In some situations, when a simulation is run for enough time, replicates may not be necessary if the simulation reached an equilibrium, by exploring most of the accessible space phase. However, the data from MD simulations have a time correlation and for that reason each point in the trajectory cannot be assumed as independent. If the independence of each frame of the trajectory was assumed, the error of a particular averaged measurement would be underestimated. For that reason, strategies like block analysis have emerged to solve the data correlation. Flyvbjerg and Petersen were probably the first to report this kind of analysis.[1] Their initial formulation was subsequently reinterpreted as described for example in ref.[2,3] .

The original idea of Flyvbjerg and Petersen was to transform the original data with size n and to reduce the data by half in each iteration until n reaches 2. In each iteration the estimated error is calculated and eventually a plateau will be reached where the estimated error will no longer vary and, at this point, a reliable error estimate is obtained. For example, an MD trajectory with a sample size of n (corresponding to the number of output frames) can be represented by the data set of each sampling point as: $x_1, x_2, x_3, \ldots, x_n$. In principle, the ensemble average of the observable o could be obtained by:

$$\langle o \rangle \equiv \int P(x) o(x)\, \mathrm{d}x \quad (1)$$

where the integral corresponds to the weighted sum over all accessible configurations in the system of the observable $o(x)$, weighted by the probability density function $P(x)$. However, in practice, we can only obtain the time average of the observable ($\bar{o}$):

$$\bar{o} = \frac{1}{n} \sum_{i=1}^{n} o(x_i) \quad (2)$$

Assuming ergodicity, meaning that the simulation was run for a sufficient amount of time and has explored all accessible states of the system:

$$\langle o \rangle = \lim_{n \to \infty} \bar{o} \quad (3)$$

the ensemble average is obtained from the time average of the observable ($\bar{o}$). However, the simulation is run for a finite time. Nevertheless, if the simulation has reached an equilibrium, the finite time average ($\bar{o}$) is a good estimator for the ensemble average. The average value (m) is then calculated as:

$$m \equiv \bar{x} = \frac{1}{n} \sum_{i=1}^{n} x_i \quad (4)$$

and the estimator for the variance of m will be:

$$\sigma^2 = \langle m^2 \rangle - \langle m \rangle^2 \quad (5)$$

From the Flyvbjerg and Petersen article[1] it was demonstrated that:

$$\sigma^2(m) \geq \langle \frac{c_0}{n - 1} \rangle \quad (6)$$

where c_0 is obtained by the following equation assuming $t = 0$:

$$c_t = \frac{1}{n - t}\sum_{i}^{n - 1}(x_i - \bar{x})(x_{i+t} - \bar{x}) \quad (7)$$

For the original data set, $c_0 / (n - 1)$ is used as the initial estimator of $\langle c_0 / (n - 1)\rangle$ in order to obtain σ^2. Now transforming the original data set into half ($n/2$ for even n, or $(n-1)/2$ for odd n), the new data values are recalculated as:

$$x'_i = \frac{1}{2}(x_{2i - 1} + x_{2i}) \quad (8)$$

where the quantities that we are trying to calculate are:

$$m' = m\ , \quad (9)$$

$$\sigma^2(m') = \sigma^2(m) \quad (10)$$

In the article,[1] these quantities were demonstrated to be invariant upon transformation of the data set. With this new data set, $\sigma^2(m')$ is again estimated through $c'_0/(n' - 1)$. Subsequently, these new data set is again transformed:

$$x''_i = \frac{1}{2}(x'_{2i-1} + x'_{2i}) \quad (11)$$

and $\sigma^2(m'')$ is again estimated through $c''_0/(n'' - 1)$. This process is repeated until n is equal to 2.

This iteration process will recalculate sequentially a new generic $\sigma^2(m')$ value. The sequential values will continuously increase until a plateau is reached, and at this constant value, $\sigma^2(m')$ will be our estimator for $\sigma^2(m)$. In the article it is shown that at the plateau of $\sigma^2(m')$, the data values x'_1, x'_2, x'_3, …, x'_n are considered independent gaussian variables making possible to estimate the standard deviation of the $\sigma^2(m)$ estimator as:

$$\sigma^2(m) = \frac{c'_0}{n'-1} \pm \sqrt{\frac{2}{n'-1} \times \frac{c'_0}{n' - 1}}\ , \quad (12)$$

$$\sigma(m) = \sqrt{\frac{c'_0}{n' - 1}} \pm \frac{1}{\sqrt{2(n' - 1)}} \quad (13)$$

If the plateau for $\sigma(m')$ is not reached before $n' = 2$, this indicates that the largest $\sigma(m')$ obtained is a lower limit of $\sigma(m)$. We realized, empirically, with this formulation, it was difficult to reach the desired plateau. For that reason, we used the formulation presented in other ref.[2,3]. The method can simply be described as the division of the trajectory into blocks with a time length equal to n, where the observable averaged value is calculated in each individual block. The blocked standard error (*BSE*) for the blocks with the length equal to n is then calculated using the averaged value of each block by:

$$BSE(o,n) = \frac{\sigma_n}{\sqrt{nB}} \quad (14)$$

where nB is the number of blocks in which the full trajectory was divided by with length equal to n, and σ_n is the estimation of the standard deviation of the averaged values obtained for each block. In each iteration, n is increased, for example by one, starting from $n = 1$, and consequently the number of blocks decreases. For each of these iterations the $BSE(o,n)$ value is calculated. The idea behind this method is based on the fact that with the increase of n, eventually, the blocks will be of sufficient size that they become statistically independent from each other. When this happens, the increase of n will not change the value of $BSE(o,n)$ and a plateau is reached. At this stage the $BSE(o,n)$ is a good estimator of the standard error of the observable, $SE(o)$. With this interpretation of the block analysis method, it was easier for us to identify the plateau than with the Flyvbjerg and Petersen method.[1] The confidence interval of the observable can then be calculated by:

$$\bar{f} \pm SE(o).t^{\alpha} \quad (15)$$

where α is the significance level. In this case we used the t student distribution, since a low number of samples were acquired. The t^{α} can be obtained from statistical tables taking into consideration the number of independent blocks estimated as the number of degrees of freedom minus 1 and at a 95% of confidence. The number of independent blocks (nB_{ind}) can be estimated by the minimum length (n) required *per* block to obtain a constant $BSE(o,n)$ and consequently the number of blocks possible to obtained from the simulated trajectory with that time length.

Appendix S.I.2 Error estimation for a weighted mean

In the present work, we used several segments of the simulated trajectories with different sampling times in order to calculate some property. For that reason, these properties were estimated through the calculation of a weighted mean, by using the average value for each time segment (x_i) and the weights as the simulated time in that segment (w_i) by the following equation:

$$\bar{x}_{\mathrm{w}} = \frac{1}{\sum_i^n w_i} \sum_{i=1}^{n} w_i x_i \quad (16)$$

The standard error of the weighted mean (SEM_{w}) was shown by Endlich *et al.*[4] and validated by Gatz and Smith[5] that it can be obtained through the ratio variance formulated by Cochran[6] as an approximation by:

$$(SEM_{\mathrm{w}})^2 = \frac{n}{(n-1)(\sum_{i=1}^{n} w_i)^2} \left[\sum_{i=1}^{n} (w_i x_i - \bar{w}\bar{x}_{\mathrm{w}})^2 - 2\bar{x}_{\mathrm{w}} \sum_{i=1}^{n} (w_i - \bar{w})(w_i x_i - \bar{w}\bar{x}_{\mathrm{w}}) + \bar{x}_{\mathrm{w}}^{2} \sum_{i=1}^{n} (w_i - \bar{w})^2 \right] \quad (17)$$

The approximated confidence interval of the weighted mean proposed by Gatz and Smith[7] is simply:

$$\bar{x}_{\mathrm{w}} \pm SEM_{\mathrm{w}} t^{\alpha} \quad (18)$$

where α represents the significance level. In the present case the t student distribution was used, since it was obtained a low number of samples, instead of the normal distribution that appears in the article of the authors.

Appendix S.I.3 Partition coefficient calculation

The interaction of solutes with lipid membranes can be described by the equilibrium:

$$\text{solute}_{(w)} \rightleftharpoons \text{solute}_{(mem)}$$

The partition coefficient (K_P) is related with the equilibrium rate constants, and is usually defined as the ratio of the local solute concentration in the membrane ($[\text{solute}]_{mem}$) and in the aqueous medium ($[\text{solute}]_w$):

$$K_P = \frac{[\text{solute}]_{mem}}{[\text{solute}]_w} \quad (19)$$

These local concentrations may be calculated from the probability density function, leading to:

$$K_P = \frac{p(\text{solute}_{(mem)}) \times \langle z_w \rangle}{p(\text{solute}_{(w)}) \times \langle z_{mem} \rangle} \quad (20)$$

where $p(\text{solute}_{(mem)})$ and $p(\text{solute}_{(w)})$ are the overall estimated probabilities to find the solute in the membrane and in the water medium, $\langle z_w \rangle$ and $\langle z_{mem} \rangle$ are the estimated width of the water and membrane slabs in the *z* coordinate, respectively. In practical terms, the latter corresponds to the location of the water/membrane interface ($z_{w/mem}$).

Traditionally,[8–10] K_P is obtained from a calculated free energy profile (FEP) along a collective variable (CV). This CV is normally defined by the *z* distance of the solute from the membrane center of mass (COM). The K_P is then determined by:

$$K_P = \frac{1}{z_{w/mem}} \int_0^{z_{w/mem}} \exp(-\Delta\Delta G(z)/RT)\, dz \quad (21)$$

where $\Delta\Delta G(z)$ corresponds to the Gibbs free energy for the solute transfer from bulk water to a specific location in the membrane along the *z* axis, and is given by:

$$\Delta\Delta G(z) = \Delta G(z) - \Delta G(z_w) \quad (22)$$

Equation 21 is described for one monolayer but is also valid for the full membrane, changing the lower integration limit to $-z_{w/mem}$ and dividing by the full bilayer thickness, $2z_{w/mem}$, instead of half its value. Because of bilayer symmetry, the integral over the whole bilayer is double that over a single bilayer, and, following division by $2z_{w/mem}$, the same result is obtained.

Although equations 20 and 21 represent two different methods to obtain K_P, they are in fact equivalent. To prove this statement, equation 21 can be rewritten as:

$$K_P = \frac{bw}{z_{w/mem}} \sum_{z=0}^{nbins_{mem}} \exp(-\Delta\Delta G(z)/RT) \quad \text{, where } nbins_{mem} = bins/\text{nm} \times z_{w/mem} \quad (23)$$

where bw represents the bin size used to obtain the probability density function, $nbins_{mem}$ corresponds to the total number of bins from the membrane COM (z=0) until $z_{w/mem}$ and $bins$/nm is simply the number of bins *per* nanometer.

The FEP along a CV can be calculated from the probability density function by:

$$\Delta G(z) = -RT \ln\left(\frac{p(z)}{p(z_{eq})}\right) \quad (24)$$

where R is the gas constant, T is the temperature and $\Delta G(z)$ is the Gibbs free energy along the CV. $p(z)$ is the estimated probability density at the z position along the CV and $p(z_{eq})$ is the estimated maximal probability density obtained along the CV. $\Delta\Delta G(z)$ from equation 22 can then be rewritten in terms of probabilities:

$$\Delta\Delta G(z) = -RT \ln\left(\frac{p(z)}{p(z_{eq})}\right) - \left(-RT \ln\left(\frac{p(z_{w/mem})}{p(z_{eq})}\right)\right) \quad (25)$$

where $p(z_{w/mem})$ corresponds to the estimated probability density of the solute at the water/membrane interface, where the FEP becomes constant, and in bulk water region. The last equation can then be simplified to:

$$\Delta\Delta G(z) = RT \ln\left(\frac{p(z_{w/mem})}{p(z)}\right) \quad (26)$$

Using the previous equation together with equation (23), we obtain:

$$K_P = \frac{bw}{z_{w/mem}} \sum_{z=0}^{nbins_{mem}} \exp\left(-RT \ln\left(\frac{p(z_{w/mem})}{p(z)}\right)/RT\right) \quad (27)$$

which can be simplified to:

$$K_P = \frac{bw}{z_{w/mem}} \sum_{z=0}^{nbins_{mem}} \frac{p(z)}{p(z_{w/mem})} \quad (28)$$

Solving the summation over the $nbins_{mem}$, the previous equation then becomes:

$$K_P = \frac{1}{z_{w/mem}} \frac{p(\text{solute}_{(mem)})}{p(z_{w/mem})} \quad (29)$$

recovering the overall estimated probability to find the solute in the membrane ($p(\text{solute}_{(mem)})$).

In order to match the previous equation with equation (20), the overall estimated probability to find the solute in water $p(\text{solute}_{(w)})$ needs to be obtained. For that purpose, we just need to multiply $p(z_{w/mem})$ by the bin width, the size in z of the water slab, and the number of bins/nanometer:

$$K_P = \frac{1}{z_{w/mem}} \frac{p(\text{solute}_{(mem)}).bw.nbins/\text{nm} .\langle z_w \rangle}{bw.nbins/\text{nm}.\langle z_w \rangle.p(z_{w/mem})} \quad ,$$

$$\text{where } p_{\text{solute}(w)} = bw.nbins/\text{nm}.\langle z_w \rangle.p(z_{w/mem}) \quad (30)$$

In practice, for the estimated $p_{solute(w)}$ calculated in this way and the one calculated in equation (20) to match, a careful choice of $\langle z_w \rangle$ needs to be done. This is due to the use of *NPT* ensemble where the system does not have a fixed volume which makes the estimated probability density artificially low near the boundaries of the systems. Since:

$$bw.nbins/\mathrm{nm}.\langle z_w \rangle = \langle z_w \rangle \quad \text{and} \quad z_{w/mem} = \langle z_{mem} \rangle \quad (31)$$

Equation 30 can then be simplified to:

$$K_P = \frac{p(\mathrm{solute}_{(mem)}) \times \langle z_w \rangle}{p(\mathrm{solute}_{(w)}) \times \langle z_{mem} \rangle} \quad (32)$$

In this way we recover equation 20, and the equivalence between equation 20 and equation 21 is shown.

Appendix S.I.4 Relaxivity theory

The relaxation of water protons in dilute solutions of paramagnetic complexes like $[Gd(DOTA)(H_2O)]^-$ is generally described by the theory of Solomon-Bloembergen-Morgan (SBM) and others.[11,12] The observed increase of the solvent proton longitudinal and transverse relaxation rates, $r_1 = \frac{1}{T_{1,\text{obs}}}$ and $r_2 = \frac{1}{T_{2,\text{obs}}}$, respectively, can be described by:

$$\left(\frac{1}{T_{i,\text{obs}}}\right) = \left(\frac{1}{T_{i,\text{p}}}\right) + \left(\frac{1}{T_{i,\text{d}}}\right) \quad i = 1,2 \qquad (33)$$

where $\frac{1}{T_{i,\text{p}}}$ and $\frac{1}{T_{i,\text{d}}}$ are the paramagnetic and diamagnetic (pure water) relaxation rates, respectively. The paramagnetic term, representing the relaxation rate enhancement due to the presence of a paramagnetic species is directly proportional to its concentration, $[Gd^{3+}]$:

$$\left(\frac{1}{T_{i,\text{obs}}}\right) = r_i\left[Gd^{3+}\right] + \left(\frac{1}{T_{i,\text{d}}}\right) \quad i = 1,2 \qquad (34)$$

where r_i (in units of $mM^{-1}\,s^{-1}$) are the water proton relaxivities induced by the paramagnetic complex, representing the relaxation rate enhancement *per* mM concentration of Gd^{3+}. With Gd^{3+}-based contrast agent (GBCA) T_1-weighted images are the relevant modality. This modality is based on the spin-lattice relaxation time (T_1) of the water protons.

The overall paramagnetic relaxation rate enhancement of the water protons, can in general, be represented by several contributions:

$$\left(\frac{1}{T_{1,\text{p}}}\right) = \left(\frac{1}{T_{1,\text{p}}}\right)^{IS} + \left(\frac{1}{T_{1,\text{p}}}\right)^{2nd} + \left(\frac{1}{T_{1,\text{p}}}\right)^{OS} \qquad (35)$$

or expressed in terms of relaxivities:

$$r_1 = r_1^{IS} + r_1^{2nd} + r_1^{OS} \qquad (36)$$

where *IS*, *2nd* and *OS* indicate the inner, second and outer-sphere terms, respectively.

The inner-sphere paramagnetic longitudinal relaxation rate is described by:

$$\left(\frac{1}{T_{1,\text{p}}}\right)^{IS} = \frac{cq^{IS}}{55.55}\cdot\frac{1}{T_{1\text{m}} + \tau_\text{m}^{IS}} \qquad (37)$$

where c is the molal concentration of the paramagnetic chelate, q is the number of water molecules coordinated in its inner-sphere, each with a residence lifetime of τ_m^{IS}, and $T_{1\text{m}}$ corresponds to the longitudinal proton relaxation time of the inner-sphere water molecules. Additionally, the longitudinal inner-sphere relaxation rate correlates with its relaxivity through:

$$\left(\frac{1}{T_{1,\text{p}}}\right)^{IS} = r_1^{IS}\left[Gd^{3+}\right] \qquad (38)$$

The longitudinal relaxation rate can be described by a scalar (through bonds) and dipolar (through space) interactions between the electronic (S) and nuclear (I) spins by:

$$\frac{1}{T_{1\mathrm{m}}} = \frac{1}{T_1^{\mathrm{DD}}} + \frac{1}{T_1^{\mathrm{SC}}} \quad (39)$$

However, for Gd^{3+} complexes, the scalar contribution to the relaxivity can be neglected. This occurs because of the long distance between the Gd^{3+} metal ion and the closest protons of an inner-sphere water molecule. The dipolar mechanism is then described by the SBM theory as:

$$\frac{1}{T_{1\mathrm{m}}} = \frac{1}{T_1^{\mathrm{DD}}} = \frac{2}{15}\frac{\gamma_I^2\mu_\mathrm{B}^2 g^2}{{r_{\mathrm{GdH}}^{IS}}^6} S(S+1)\left(\frac{\mu_0}{4\pi}\right)^2\left[7\frac{\tau_{\mathrm{c}2}}{1+\omega_S^2\tau_{\mathrm{c}2}^2} + 3\frac{\tau_{\mathrm{c}1}}{1+\omega_I^2\tau_{\mathrm{c}1}^2}\right] \quad (40)$$

where γ_I is the nuclear gyromagnetic ratio (for the proton $\gamma_I = 2.675 \times 10^8$ $T^{-1}s^{-1}$), μ_B is the Bohr magneton (9.24×10^{-24} $J.T^{-1}$), g is the electron g-factor (for Gd^{3+} is equal to 2), r_{GdH} is the distance between the Gd^{3+}, the electron spin, and the proton spin (normally an assumed value of 0.31 nm, based on other works or X-ray crystallographic structures), the electronic spin S of the paramagnetic Gd^{3+} metal ion is 7/2, ω_S and ω_I are the electron and nuclear spin Larmor frequencies (at a magnetic field with strength B(T), $\omega = \gamma B$), respectively, μ_0 is the vacuum permeability ($\mu_0 = 4\pi\times10^{-7}$ TmA^{-1}), and the τ_{ci} are the correlation times, described by:

$$\frac{1}{\tau_{\mathrm{c}i}} = \frac{1}{\tau_\mathrm{R}} + \frac{1}{T_{i\mathrm{e}}} + \frac{1}{\tau_\mathrm{m}^{IS}}\ , i = 1,2 \quad (41)$$

where, in turn, τ_R is the rotational correlation time of the Gd-H_{water} vector and T_{ie} (i = 1,2) are the longitudinal and transverse relaxation times of Gd^{3+} electron spin. The electronic spin relaxation T_{1e} and T_{2e} is normally interpreted as a zero-field splitting interaction (*ZFS*):

$$\left(\frac{1}{T_{1\mathrm{e}}}\right)^{ZFS} = \frac{1}{25}\Delta^2\tau_\mathrm{v}[4S(S+1)-3]\left(\frac{1}{1+\omega_S^2\tau_\mathrm{v}^2} + \frac{4}{1+4\omega_S^2\tau_\mathrm{v}^2}\right) \quad (42)$$

$$\left(\frac{1}{T_{2\mathrm{e}}}\right)^{ZFS} = \frac{1}{50}\Delta^2\tau_\mathrm{v}[4S(S+1)-3]\left(\frac{5}{1+\omega_S^2\tau_\mathrm{v}^2} + \frac{2}{1+4\omega_S^2\tau_\mathrm{v}^2} + 3\right) \quad (43)$$

where τ_v is the *ZFS* modulation correlation time and Δ^2 is the *ZFS* mean square fluctuation.

When a metal complex interacts with a larger compound or aggregate, its rotational correlational time slows down. When this occurs, the overall rotational correlational time can be decomposed into the local (τ_l) and global (τ_g) motion that can be obtained by the Lipari-Szabo approach[13,14]. These two types of motions can have a percentage of coupling that is accounted into the generalized order parameter S (sometimes referred as F to prevent potential confusion with the electronic spin S). This approach is included into the correlational time and the spectral density function as:

$$\frac{1}{T_{1\mathrm{m}}} = \frac{1}{T_1^{\mathrm{DD}}} = \frac{2}{15}\frac{\gamma_I^2 g^2\mu_\mathrm{B}^2}{{r_{\mathrm{GdH}}^{IS}}^6} S(S+1)\left(\frac{\mu_0}{4\pi}\right)^2\left[7.\left(\frac{F^2\tau_{\mathrm{d2g}}}{1+\omega_S^2\tau_{\mathrm{d2g}}^2} + \frac{(1-F^2)\tau_{\mathrm{d2}}}{1+\omega_S^2\tau_{\mathrm{d2}}^2}\right) + 3.\left(\frac{F^2\tau_{\mathrm{d1g}}}{1+\omega_I^2\tau_{\mathrm{d1g}}^2} + \frac{(1-F^2)\tau_{\mathrm{d1}}}{1+\omega_I^2\tau_{\mathrm{d1}}^2}\right)\right] \quad (44)$$

where:

$$\frac{1}{\tau_{\mathrm{d}i\mathrm{g}}} = \frac{1}{\tau_\mathrm{g}} + \frac{1}{T_{i\mathrm{e}}} + \frac{1}{\tau_\mathrm{m}^{IS}}\ , i = 1,2 \quad (45)$$

$$\frac{1}{\tau_{\mathrm{d}i}} = \frac{1}{\tau} + \frac{1}{T_{i\mathrm{e}}} + \frac{1}{\tau_{\mathrm{m}}^{IS}} \;, i = 1{,}2 \quad (46)$$

with:

$$\frac{1}{\tau} = \frac{1}{\tau_{\mathrm{g}}} + \frac{1}{\tau_{\mathrm{l}}} \quad (47)$$

where τ_{g} corresponds to the global rotational correlational lifetime motion and τ_{l} the local rotational correlational lifetime motion.

The second-sphere term is employed to account for water molecules that remain in close proximity to the chelate by establishing interactions with its functional groups (e.g. phosphate and carboxylate groups). This term is treated in a similar manner as the inner-sphere term, but it considers all potential different binding sites:

$$\left(\frac{1}{T_{1,\mathrm{p}}}\right)^{2nd} = \frac{c}{55.55}\sum_{j=1}^{q^{2nd}} \frac{q_j^{2nd}}{T_{1\mathrm{m},j}^{2nd} + \tau_{\mathrm{m},j}^{2nd}} \quad (48)$$

For a specific binding site j, q_j^{2nd} represents the number of water molecules in that binding site, $T_{1\mathrm{m},j}^{2nd}$ corresponds to the longitudinal proton relaxation time in that binding site. In the latter term, adjustments need to be made for the new Gd^{+3}-proton distance ($r_{\mathrm{GdH},j}^{2nd}$) of the water molecules in that binding site, their residence lifetime ($\tau_{\mathrm{m},j}^{2nd}$), and the new correlation time ($\tau_{\mathrm{c},i,j}^{2nd}$). $\tau_{\mathrm{c},i,j}^{2nd}$ can then be calculated from:

$$\frac{1}{\tau_{\mathrm{c},i,j}^{2nd}} = \frac{1}{\tau_{\mathrm{R}}} + \frac{1}{T_{i\mathrm{e}}} + \frac{1}{\tau_{\mathrm{m},j}^{2nd}} \;, i = 1{,}2 \quad (49)$$

The outer-sphere represents the molecules of water diffusing in the proximity of the chelate, in a location closer than the bulk water molecules. This term is usually described by Freed's equation:

$$\left(\frac{1}{T_{1,\mathrm{p}}}\right)^{OS} = \frac{32\pi}{405}\,\frac{N_{\mathrm{A}}\left[\mathrm{Gd}^{3+}\right]}{dD_{\mathrm{GdH}}}\left(\frac{\mu_0}{4\pi}\right)^2 \gamma_I^2\gamma_S^2\hbar^2 S(S+1)\left[j_2(\omega_I - \omega_S) + 3j_1(\omega_I) + 6j_2(\omega_I + \omega_S)\right] \quad (50)$$

where N_{A} is the Avogadro constant, d represents the closest possible distance of the outer-sphere from Gd^{3+}, and D_{GdH} is the diffusion constant of the chelate plus the water protons ($D_{\mathrm{GdH}} = D_{[\mathrm{Gd(Ligand)}]} + D_{\mathrm{H_2O}}$). The latter constant is normally an assumed value, approximated by the Stokes-Einstein equation plus the experimental value of the water self-diffusion. Additionally, $\hbar$ is the reduced Planck constant and $j(\omega)$ are the spectral densities, given by:

$$j_k(\omega) = \mathrm{Re}\left\{\frac{1 + z/4}{1 + z + 4z^2/9 + z^3/9}\right\} \quad (51)$$

and,

$$\text{with } z = \sqrt{i\omega.\frac{d^2}{D_{\mathrm{GdH}}} + \frac{d^2}{D_{\mathrm{GdH}}T_{k\mathrm{e}}}} \;, \text{with } k=1{,}2 \quad (52)$$

k is used in the last equation instead of the typical *i* used in the rest of the equations, to differentiate it from the imaginary unit.

In general terms, within their approximations, the previous equations presented here explain the relaxivity induced by a paramagnetic center to the water protons. Normally, these are the equations used to fit the Nuclear Magnetic Relaxation Dispersion (NMRD) profile where the relaxivity is measured as a function of the strength of the magnetic field. Additionally, ^{17}O NMR experiments with labeled water are performed in order to reduce the number of adjustable constants in the NMRD fitting. In ^{17}O NMR experiments, the longitudinal and transverse relaxation times are measured as a function of temperature. These experiments are normally analyzed with similar equations. In the longitudinal relaxation the quadrupolar contribution, due to the ^{17}O nuclei, and the dipolar contribution are the major mechanisms. In the ^{17}O transverse relaxation, the scalar contribution is the dominant mechanism. These experiments allow the determination of τ_m and τ_R. In some situations, additional data on the electronic relaxation process is required, which are normally acquired from electron paramagnetic resonance spectroscopy (EPR) experiments. Additionally, when there are doubts in the number of inner sphere water molecules (q^{IS}), time resolved luminescence spectroscopy is performed. These experiments are normally done by the substitution of the Gd^{3+} in the complex by Eu^{3+} or Tb^{3+} metal ions, due to their different physical properties. This substitution is justified because the chemistry of the lanthanide series is essentially the same and all of them interact with the organic ligand through electrostatical interactions. In addition, their ionization state is the same and the ionic radii of Eu^{3+}, Tb^{3+} and Gd^{3+} are similar, inducing low conformational changes in the complex cage. These experiments are based on the different luminescent lifetimes due to the water/deuterium isotope effect that induces different deactivation rates of the excited state of the metal ion.

Overall, in terms of parameters needed to be adjusted in a NMRD profile, we can count: q^{IS}, r_{GdH}^{IS}, τ_R, τ_m^{IS}, Δ^2, τ_v, q_j^{2nd}, r_{GdH}^{2nd}, τ_m^{2nd} and *d*. If the Lipari-Szabo approach is used the τ_R is decomposed into local (τ_l) and global (τ_g) motion, with an additional coupling parameter (*S*) between both motions. That makes the fitting a problem involving at least 12 adjustable parameters. Although some of these parameters can be determined with other experiments, by performing those experiments, such as those previously described, adjustment of additional parameters may be required.[11,12]

Additional Figures and Tables

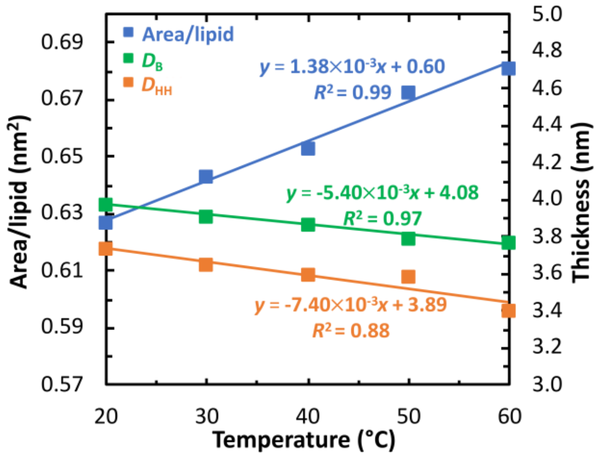

Figure S1. Area *per* lipid (blue), Luzzati thickness (D_B, green) and the distance between the phosphate groups in different monolayers (D_{HH}, orange) for a POPC lipid bilayer at different temperatures. Symbols correspond to the experimental values obtained from Kučerka *et al.* using X-ray and small-angle neutron scattering data.[15] The line corresponds to the linear regression of the experimental data.

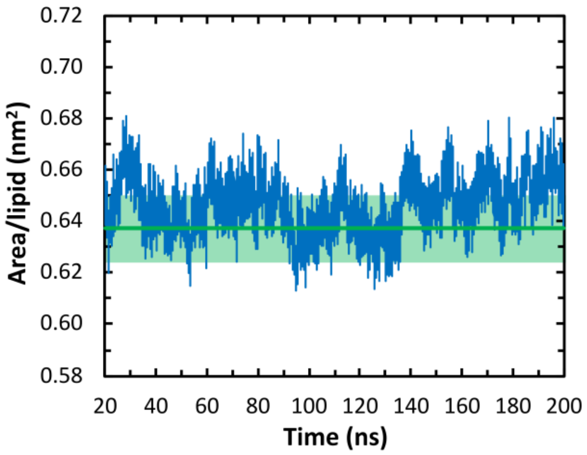

Figure S2. Area *per* lipid for a POPC bilayer using the new version of Slipids force field[16] with a cut-off for the electrostatics and van der Waals interaction of 1.4 nm. The green line represents the experimental value from Kucerka *et al.* with the green area representing experimental error reported as 0.013 nm^2.[15]

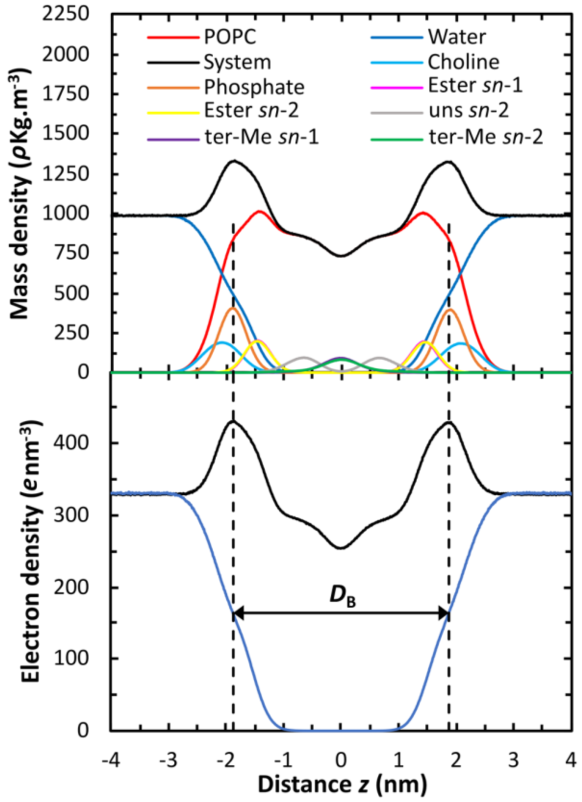

Figure S3. Mass and electron density profile for POPC membrane using the new version of Slipids force field[16] with a cut-off for the electrostatics and van der Waals interaction of 1.4 nm. In the electron density profile, the Luzzati thickness (D_B) is represented.

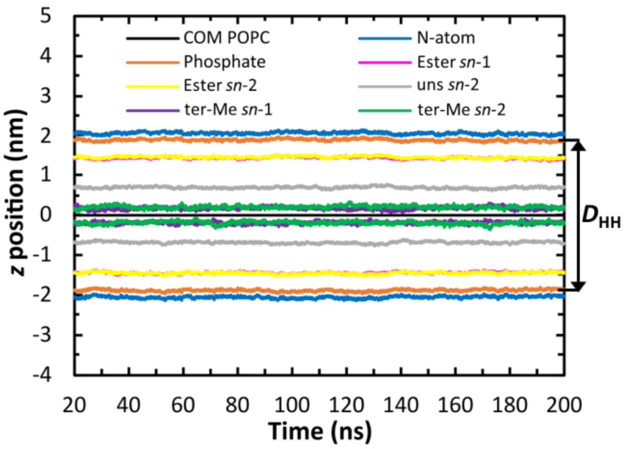

Figure S4. *z* position of different groups of the lipids compared to the COM of the POPC lipid bilayer using the new version of Slipids[16], with a cut-off for the electrostatics and van der Waals interaction of 1.4 nm. The head-to-head distance (D_{HH}) is indicated as the distance between the phosphate group planes in each monolayer.

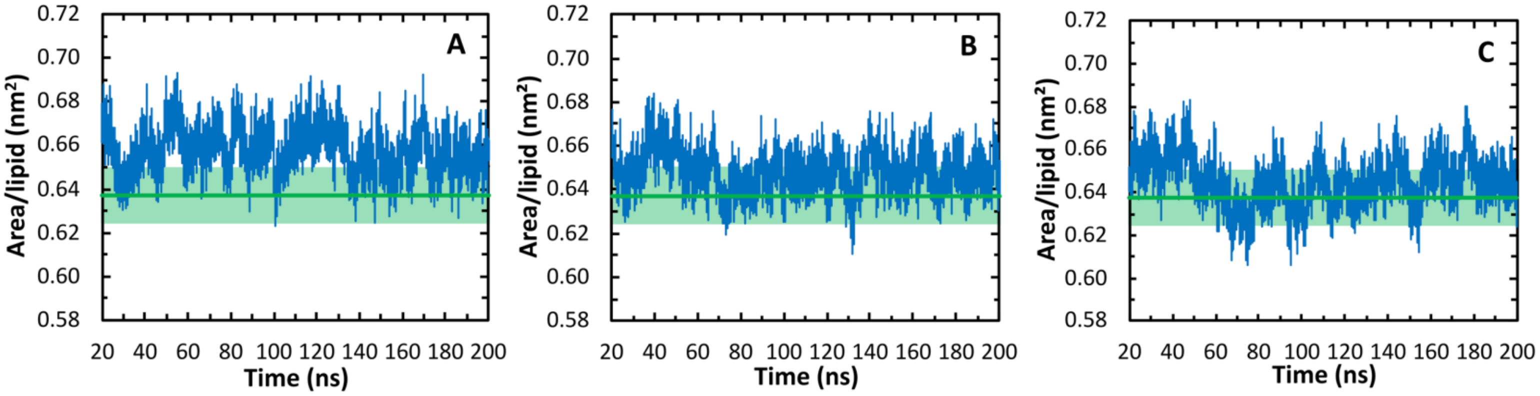

Figure S5. Area *per* lipid for a POPC bilayer using the old version of Slipids force field[17,18] with a cut-off for the electrostatics and Van der Waals interaction of 1.0 nm **(A)**, 1.2 nm **(B)** and 1.4 nm **(C)**. The green line represents the experimental value from Kucerka *et al.* with the green area representing experimental error reported as 0.013 nm^2.[15]

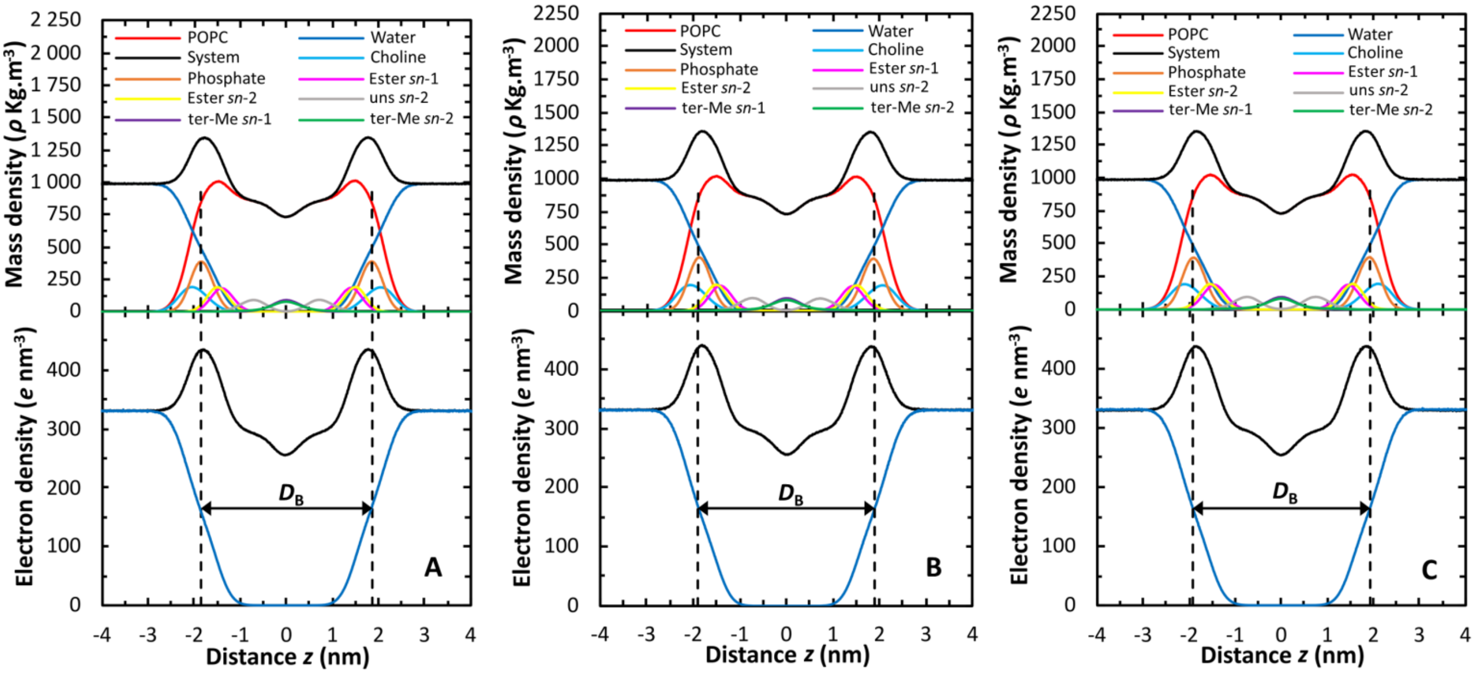

Figure S6. Mass and electron density profile for POPC membrane using the old version of Slipids force field[17,18] with a cut-off for the electrostatics and van der Waals interactions of 1.0 nm **(A)**, 1.2 nm **(B)** and 1.4 nm **(C)**. In the electron density profiles, the Luzzati thickness (D_B) is represented.

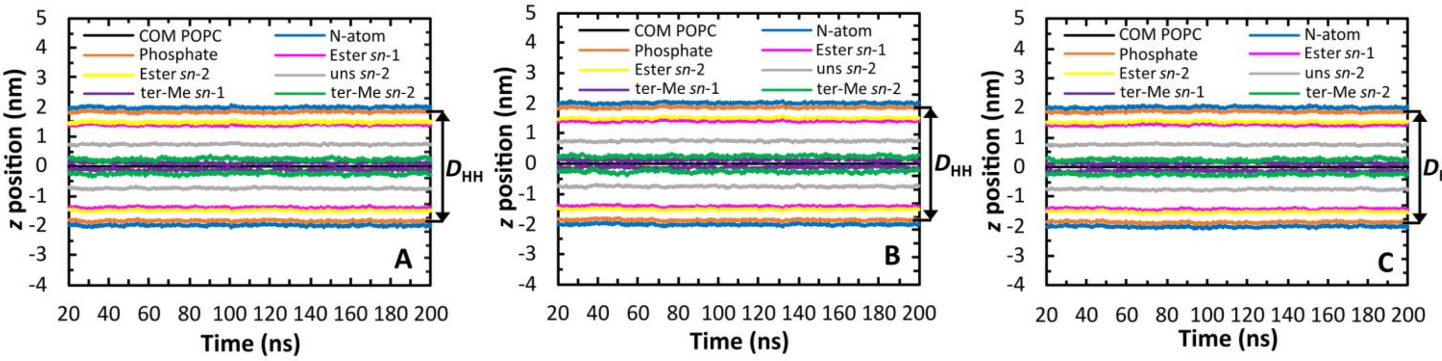

Figure S7. *z* position of different groups of the lipids compared to the COM of the POPC lipid bilayer using the old version of Slipids force field[17,18] with a cut-off for the electrostatics and van der Waals interactions of 1.0 nm **(A)**, 1.2 nm **(B)** and 1.4 nm **(C)**. The head-to-head distance (D_{HH}) is indicated as the distance between the phosphate group planes in each monolayer.

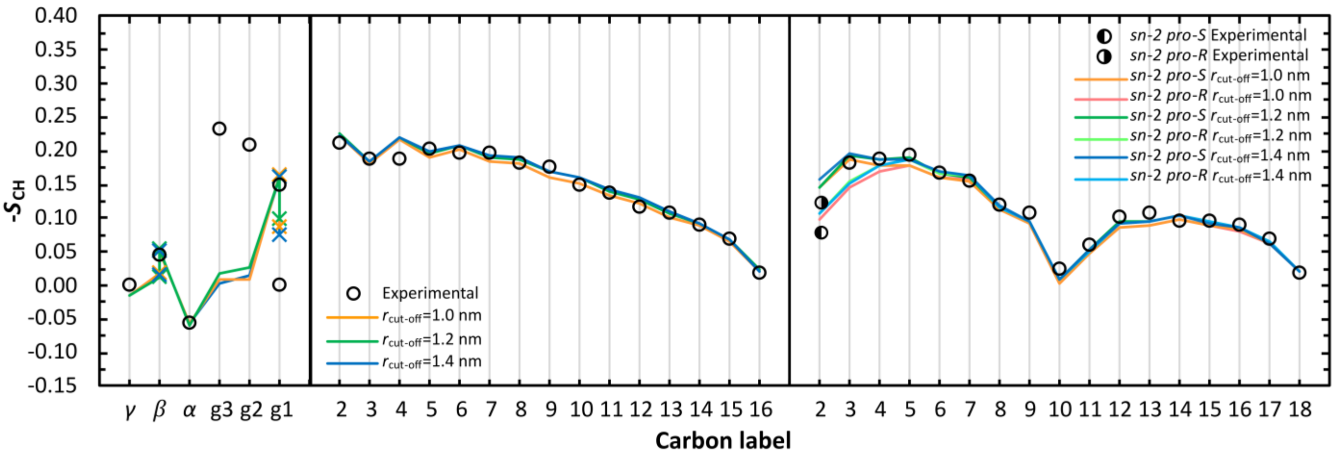

Figure S8. Order parameter ($-S_{CH}$) of the head group (**left**) the *sn*-1 tail (**middle**) and *sn*-2 tail (**right**) of POPC in the lipid bilayer using the old version of Slipids force field[17,18], with a cut-off for the electrostatics and van der Waals interactions of 1.0 nm, 1.2 nm and 1.4 nm. In the representation of the head group and *sn*-1 order parameters, splitting of the order parameter for the same carbon is only shown when the difference between the two values was over 0.02. In the calculation of the order parameters in the *sn*-2 chain, the bonds between the prochiral carbon atoms and the *pro-R* and *pro-S* hydrogen atoms were differentiated.

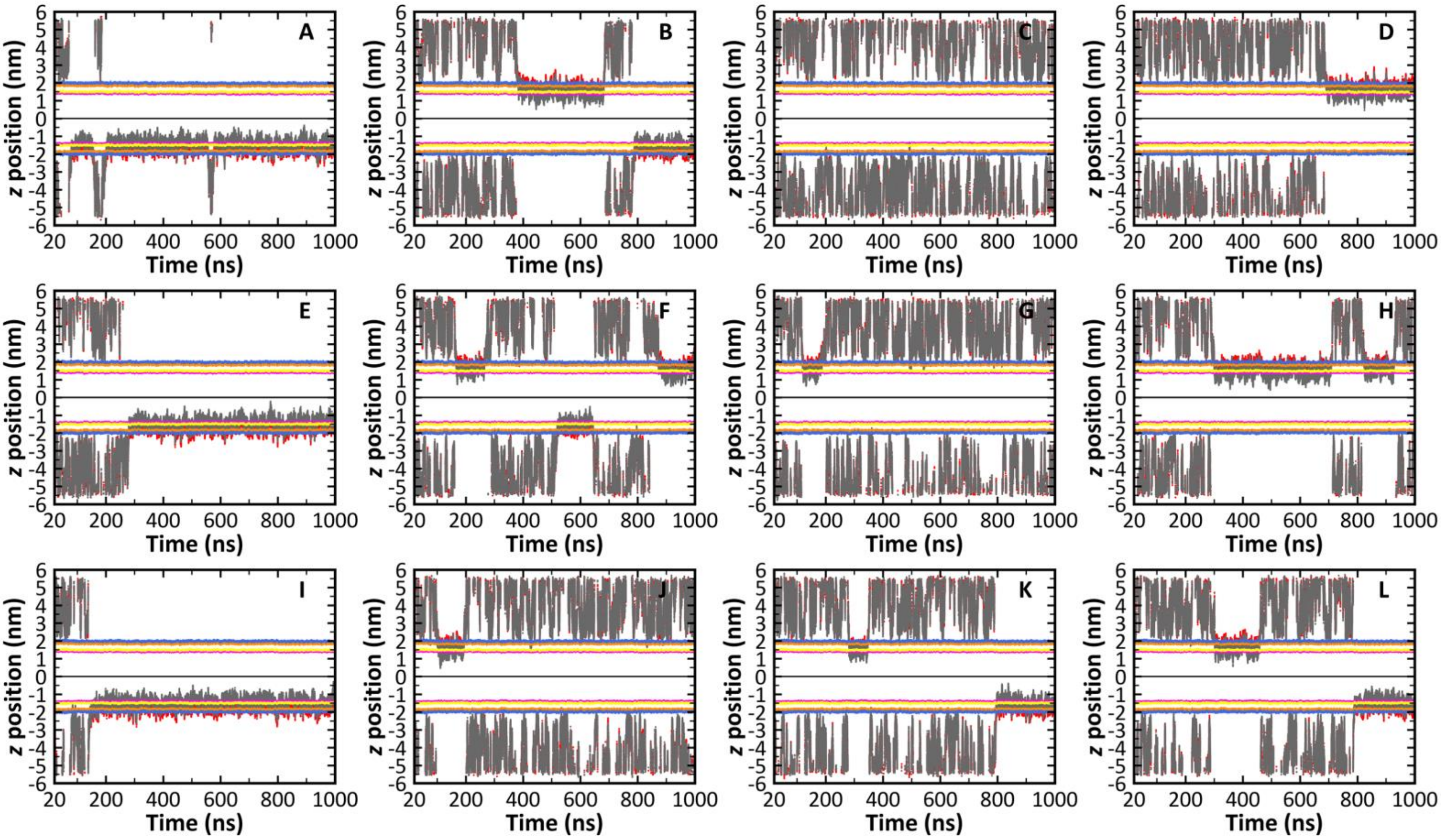

Figure S9. Trajectory of [Gd(DOTA)]$^-$ in a system with a POPC membrane. Three replicates (**A-D**, **E-H** and **I-L**) were simulated where each system contained 4 [Gd(DOTA)]$^-$ complexes that were initially positioned in the water medium (each different plot corresponds to the trajectory of a different [Gd(DOTA)]$^-$ chelate inside each system or replicate). The hydrophobic (gray line) and the hydrophilic (red line) part of [Gd(DOTA)]$^-$ (according to Figure 1 in the main text) are differentiated in each individual plot. The trajectory of specific groups of POPC are also plotted in each individual plot, namely the N atom (blue line), the phosphate group (orange line), the ester group from *sn*-1 tail (fuchsia line), the ester group from *sn*-2 tail (yellow line) and the POPC COM (black line).

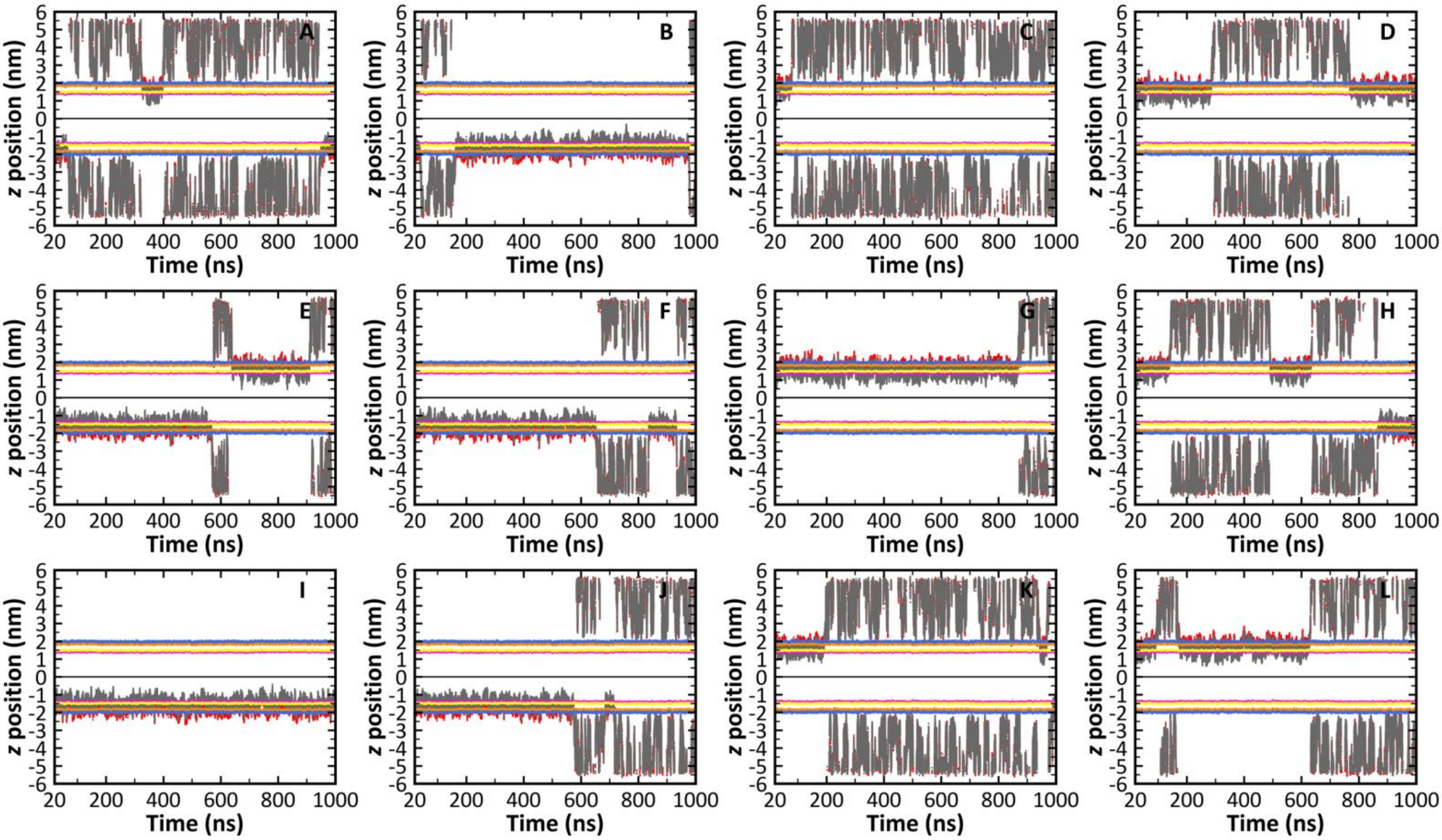

Figure S10. Trajectory of [Gd(DOTA)]$^-$ in a system with a POPC membrane. Three replicates (**A-D**, **E-H** and **I-L**) were simulated where each system contained 4 [Gd(DOTA)]$^-$ complexes that were positioned inserted in the membrane, close to the COM of the latter (each different plot corresponds to the trajectory of a different [Gd(DOTA)]$^-$ chelate inside each system or replicate). The hydrophobic (gray line) and the hydrophilic (red line) part of [Gd(DOTA)]$^-$ (according to Figure 1 in the main text) are differentiated in each individual plot. The trajectory of specific groups of POPC are also plotted in each individual plot, namely the N atom (blue line), the phosphate group (orange line), the ester group from *sn*-1 tail (fuchsia line), the ester group from *sn*-2 tail (yellow line) and the POPC COM (black line).

Table S1. Fitting parameters of the rotational correlation functions in Figure 5 from the main text. The fitted equations are of the type $C(t)= (\sum_{i=1}^{n} a_i e^{-k_i t}) + a_\infty$, where n represents the number of exponential functions required to have the correct fitting of the data.

	a_1	k_1 (ns^{-1})	a_2	k_2 (ns^{-1})	a_3	k_3 (ns^{-1})	a_4	k_4 (ns^{-1})	a_∞	τ_1 (ps)	τ_2 (ps)	τ_3 (ps)	τ_4 (ps)
**Ocoord plane (complex inserted)**	0.136	41.9	0.231	3.11	0.264	0.684	0.117	0.163	0.240	23.9	322	1461	6138
**Gd-Ow (complex inserted)**	0.114	109	0.201	6.06	0.362	0.828	0.081	0.130	0.241	9.2	165	1208	7668
**Gd-Hw (complex inserted)**	0.284	204	0.169	6.44	0.291	0.835	0.063	0.130	0.192	4.9	155	1198	7711
**Ocoord plane (complex in water)**	0.900	43.4	0.092	6.30	---	---	---	---	---	23.0	159	---	---
**Gd-Ow (complex in water)**	0.918	44.3	0.073	5.51	---	---	---	---	---	22.6	181	---	---
**Gd-Hw (complex in water)**	0.291	238	0.667	38.2	0.042	4.30	---	---	---	4.2	26	233	---

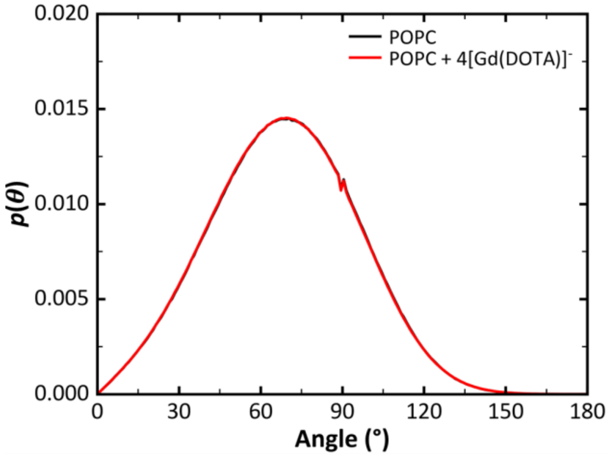

Figure S11. Probability density functions, $p(\theta)$, of the POPC P-N tilt angle, relative to the lipid bilayer normal, for the POPC only system (black line) and for the POPC with four $[Gd(DOTA)]^-$ system (red line) at 310.15 K.

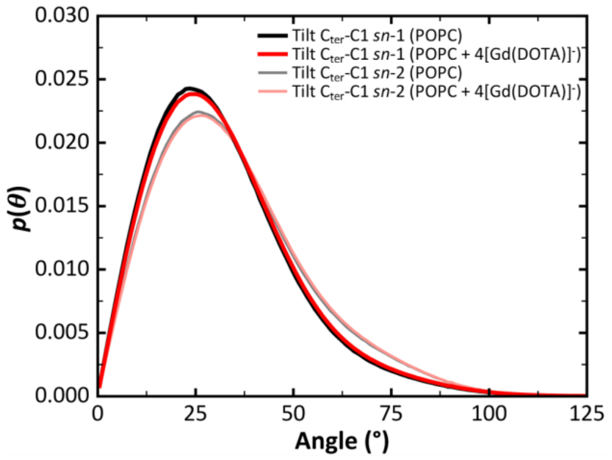

Figure S12. Probability density functions, $p(\theta)$, of the POPC C_{ter}-C1 tilt angle, relative to the lipid bilayer normal, for the *sn*-1 and *sn*-2 chains, for the POPC only system (black and gray line), and for the POPC bilayer in the presence of four $[Gd(DOTA)]^-$ chelates (red and pink line) at 310.15 K.

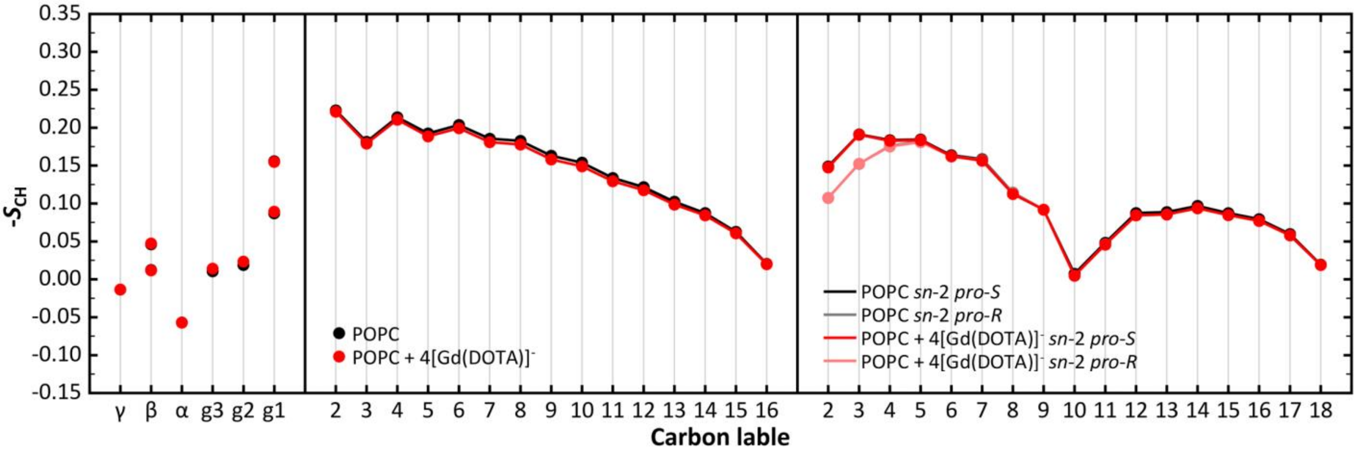

Figure S13. Order parameters $-S_{CH}$ for the POPC head group, *sn*-1 and *sn*-2 chain calculated for pure POPC system (black line) and for POPC in the presence of four [Gd(DOTA)]$^-$ chelates (red line) at 310.15 K. Regarding the calculation of the order parameters in the *sn*-2 chain, the bonds between each prochiral carbon atoms and the *pro-S* and *pro-R* hydrogen atoms were differentiated.

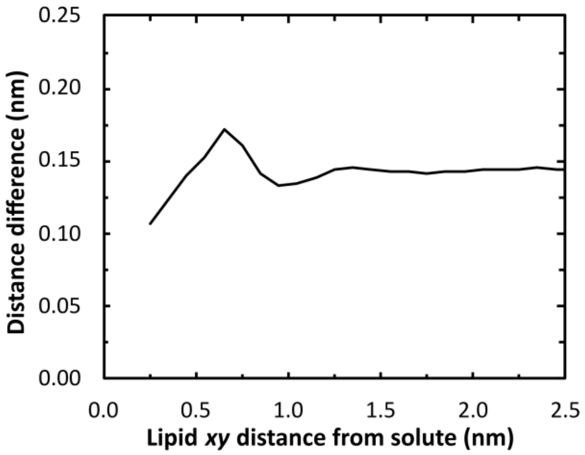

Figure S14. Vertical difference between the relative positions of N and P atoms of POPC as a function of the *xy* distance from the [Gd(DOTA)]$^-$. The *xy* distances are calculated from the COM of the [Gd(DOTA)]$^-$ to the phosphorous atom of the POPC molecules.

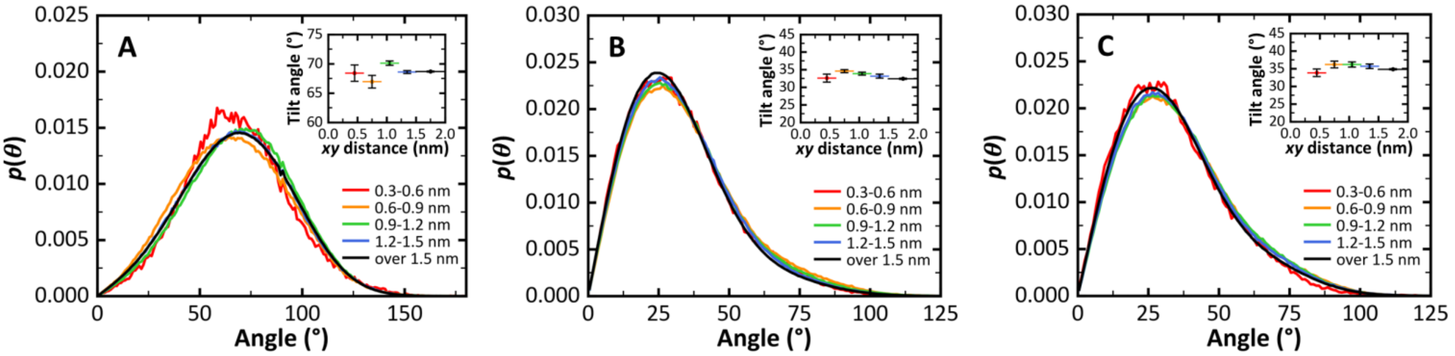

Figure S15. Probability density functions, $p(\theta)$, of the P-N tilt angle of POPC (**A**) and of the tilt angle of the vector C_{ter}-C1 of the *sn*-1 (**B**) and *sn*-2 (**C**) chain of POPC, relative to the lipid bilayer normal. The probability density functions are calculated for five bins that correspond to different *xy* distances of POPC molecules from $[Gd(DOTA)]^-$. The inserted plot represents the bin average values of those probability density functions. The colors in the inserted plot match the colors of the probability density functions.

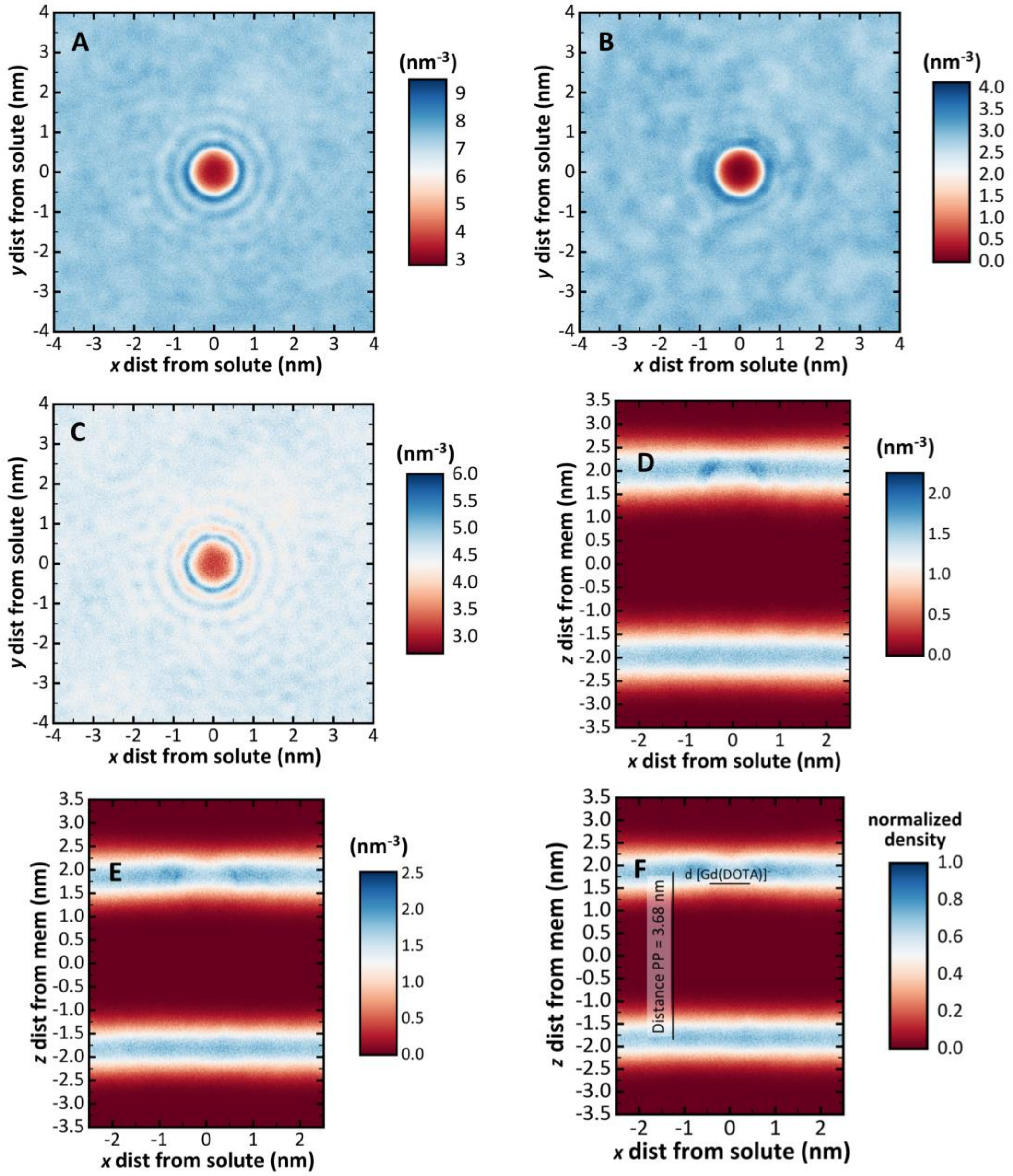

Figure S16. Density maps in real values (nm^{-3}) as a function of the distance from the $[Gd(DOTA)]^-$ COM to all heavy atoms of POPC (**A**), to the POPC head group heavy atoms (corresponding to the atoms ranging from the choline group to the ester groups of the acyl chains including the C1 carbon) (**B**) and to all heavy atoms of the *sn*-1 and *sn*-2 acyl chains (corresponding to the carbon atoms from C2 to C_{ter} atoms) (**C**). These density maps were obtained for the *x* and *y* planes averaging over the *z* plane. Density maps in real values (nm^{-3}) as a function of the *x* distance of the POPC from the $[Gd(DOTA)]^-$ COM and the *z* distance from the membrane COM for the N (**D**) and P (**E**) atoms of POPC. These density maps were obtained for the *x* and *z* planes averaging over the *y* plane. Normalized density map of the P atoms of POPC (**F**). In this map, the real location of the $[Gd(DOTA)]^-$ and the distance between the P atoms plane of each monolayer are indicated. All the density maps correspond to the trajectory of the $[Gd(DOTA)]^-$ in Figure S10I. Additionally, the three latter density maps were rotated in order for the lower monolayer to be represented in the upper part of the figure for better visualization.

Table S2. Fitting parameters of the RDF profiles from Figure 7 in the main text. The fitting of the RDF profile of all POPC heavy atoms around all POPC heavy atoms are based on a sinusoidal damped function with the form: $f(x) = A \times \exp(-k_a x) \times \cos(\omega x + \alpha) + C$). Concerning the RDF around the COM of $[Gd(DOTA)]^-$, it was only possible to fit the RDF profile after the first peak. Besides the typical sinusoidal damped function described above, the C parameter that refers to the straight line around which the oscillations occur was modified to account for the slowing increase of the oscillation node. This term is based on a logistic function with the form: $f(x)=\frac{L}{1 + \exp(-k_L \times (x - x_0))}$.

Fitting curve	*A*	k_a	*ω*	*α*	*C*	*L*	k_L	x_0
**RDF POPC-POPC**<br>$f(x) = A \times \exp(-k_a x) \times \cos(\omega x + \alpha) + C$	0.13	2.55	14.09	-0.78	1.00	-	-	-
**RDF $[Gd(DOTA)]^-$-POPC**<br>$f(x) = A \times \exp(-k_a x) \times \cos(\omega x + \alpha) + \frac{L}{1 + \exp(-k_L \times (x - x_0))}$	0.35	2.00	13.15	-1.90	-	1.01	0.86	-3.90

Table S3. Position in nanometers of the most relevant peaks presented in the RDF profiles of the Figure 7, 8A, 8B, 8C, 8D, 8D, 8E, 8F, 8G and 9b of the main text.

		1st peak position (nm)	2nd peak position (nm)	3rd peak position (nm)	4th peak position (nm)
**Figure 7**	RDF POPC – POPC	0.488	0.936		
	RDF $[Gd(DOTA)]^-$ – POPC	0.690	1.090	1.568	
**Figure 8A**	RDF Gd – O_{water} ($[Gd(DOTA)]^-$ inserted)	0.241	0.451	0.665	
	RDF Gd – H_{water} ($[Gd(DOTA)]^-$ inserted)	0.305	0.373		
	RDF Gd – O_{water} ($[Gd(DOTA)]^-$ in water)	0.242	0.458	0.676	
	RDF Gd – H_{water} ($[Gd(DOTA)]^-$ in water)	0.308	0.377		
**Figure 8B**	RDF non-coord O – O_{water} ($[Gd(DOTA)]^-$ inserted)	0.274	0.398		
	RDF non-coord O – H_{water} ($[Gd(DOTA)]^-$ inserted)	0.180	0.306		
	RDF non-coord O – O_{water} ($[Gd(DOTA)]^-$ in water)	0.276	0.404		
	RDF non-coord O – H_{water} ($[Gd(DOTA)]^-$ in water)	0.180	0.308		
**Figure 8C**	RDF coord O – O_{water} ($[Gd(DOTA)]^-$ inserted)	0.281			
	RDF coord O – H_{water} ($[Gd(DOTA)]^-$ inserted)	0.178	0.309	0.369	
	RDF coord O – O_{water} ($[Gd(DOTA)]^-$ in water)	0.280			
	RDF coord O – H_{water} ($[Gd(DOTA)]^-$ in water)	0.178	0.312	0.372	
**Figure 8D**	RDF non-coord O – Na^+ ($[Gd(DOTA)]^-$ inserted)	0.238	0.467		
	RDF non-coord O – Na^+ ($[Gd(DOTA)]^-$ in water)	0.240	0.470		
**Figure 8E**	RDF Gd – Phosphate	0.837			
**Figure 8F**	RDF non-coord O – Choline	0.221	0.318	0.363	0.553
**Figure 8G**	RDF Gd – $O_{carbonyl}$ Ester *sn*-1	0.681	0.877		
	RDF Gd – $O_{carbonyl}$ Ester *sn*-2	0.678	0.891		
**Figure 9B**	$C_{methylene}$ – $O_{carbonyl}$ *sn*-1	0.366			
	$C_{methylene}$ – $O_{carbonyl}$ *sn*-2	0.356			
	$H_{methylene}$ – $O_{carbonyl}$ *sn*-1	0.289			
	$H_{methylene}$ – $O_{carbonyl}$ *sn*-2	0.278			

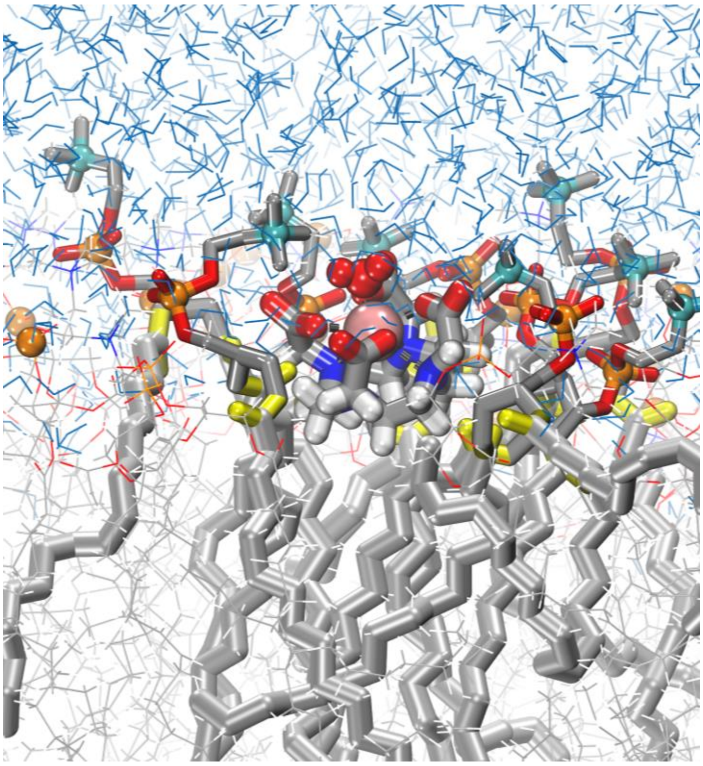

Figure S17. Expanded snapshot presented in Figure 8H of the main text with higher resolution. Additional information about the snapshot can be found in the caption of this figure in the main text.

References

(1) Flyvbjerg, H.; Petersen, H. G. Error Estimates on Averages of Correlated Data. *J. Chem. Phys.* **1989**, *91* (1), 461–466. https://doi.org/10.1063/1.457480.

(2) Grossfield, A.; Zuckerman, D. M. Chapter 2 Quantifying Uncertainty and Sampling Quality in Biomolecular Simulations. In *Annual Reports in Computational Chemistry*; Elsevier, 2009; Vol. 5, pp 23–48. https://doi.org/10.1016/S1574-1400(09)00502-7.

(3) Grossfield, A.; Patrone, P. N.; Roe, D. R.; Schultz, A. J.; Siderius, D.; Zuckerman, D. M. Best Practices for Quantification of Uncertainty and Sampling Quality in Molecular Simulations [Article v1.0]. *Living J. Comput. Mol. Sci.* **2019**, *1* (1), 1–24. https://doi.org/10.33011/livecoms.1.1.5067.

(4) Endlich, R. M.; Eymon, B. P.; Ferek, R. J.; Valdes, A. D.; Maxwell, C. Statistical Analysis of Precipitation Chemistry Measurements over the Eastern United States. Part I: Seasonal and Regional Patterns and Correlations. *J. Appl. Meteorol.* **1988**, *27* (12), 1322–1333. https://doi.org/10.1175/1520-0450(1988)027<1322:SAOPCM>2.0.CO;2.

(5) Gatz, D. F.; Smith, L. The Standard Error of a Weighted Mean Concentration—I. Bootstrapping vs Other Methods. *Atmos. Environ.* **1995**, *29* (11), 1185–1193. https://doi.org/10.1016/1352-2310(94)00210-C.

(6) G.Cochran, W. *Sampling Techniques*, third edit.; John Wiley and Sons Inc: New York, 1977.

(7) Gatz, D. F.; Smith, L. The Standard Error of a Weighted Mean Concentration—II. Estimating Confidence Intervals. *Atmos. Environ.* **1995**, *29* (11), 1195–1200. https://doi.org/10.1016/1352-2310(94)00209-4.

(8) MacCallum, J. L.; Tieleman, D. P. Computer Simulation of the Distribution of Hexane in a Lipid Bilayer: Spatially Resolved Free Energy, Entropy, and Enthalpy Profiles. *J. Am. Chem. Soc.* **2006**, *128* (1), 125–130. https://doi.org/10.1021/ja0535099.

(9) Magalhães, N.; Simões, G. M.; Ramos, C.; Samelo, J.; Oliveira, A. C.; Filipe, H. A. L.; Ramalho, J. P. P.; Moreno, M. J.; Loura, L. M. S. Interactions between Rhodamine Dyes and Model Membrane Systems—Insights from Molecular Dynamics Simulations. *Molecules* **2022**, *27* (4), 1420. https://doi.org/10.3390/molecules27041420.

(10) Kiametis, A. S.; Stock, L.; Cirqueira, L.; Treptow, W. Atomistic Model for Simulations of the Sedative Hypnotic Drug 2,2,2-Trichloroethanol. *ACS Omega* **2018**, *3* (11), 15916–15923. https://doi.org/10.1021/acsomega.8b02017.

(11) Merbach, A.; Helm, L.; Tóth, É. *The Chemistry of Contrast Agents in Medical Magnetic Resonance Imaging*, 2nd ed.; Wiley, 2013.

(12) Wahsner, J.; Gale, E. M.; Rodríguez-Rodríguez, A.; Caravan, P. Chemistry of MRI Contrast Agents: Current Challenges and New Frontiers. *Chem. Rev.* **2019**, *119* (2), 957–1057. https://doi.org/10.1021/acs.chemrev.8b00363.

(13) Lipari, G.; Szabo, A. Model-Free Approach to the Interpretation of Nuclear Magnetic Resonance Relaxation in Macromolecules. 1. Theory and Range of Validity. *J. Am. Chem. Soc.* **1982**, *104* (17), 4546–4559. https://doi.org/10.1021/ja00381a009.

(14) Lipari, G.; Szabo, A. Model-Free Approach to the Interpretation of Nuclear Magnetic Resonance Relaxation in Macromolecules. 2. Analysis of Experimental Results. *J. Am. Chem. Soc.* **1982**, *104* (17), 4559–4570. https://doi.org/10.1021/ja00381a010.

(15) Kučerka, N.; Nieh, M.-P.; Katsaras, J. Fluid Phase Lipid Areas and Bilayer Thicknesses of Commonly Used Phosphatidylcholines as a Function of Temperature. *Biochim. Biophys. Acta - Biomembr.* **2011**, *1808* (11), 2761–2771. https://doi.org/10.1016/j.bbamem.2011.07.022.

(16) Grote, F.; Lyubartsev, A. P. Optimization of Slipids Force Field Parameters Describing Headgroups of Phospholipids. *J. Phys. Chem. B* **2020**, *124* (40), 8784–8793. https://doi.org/10.1021/acs.jpcb.0c06386.

(17) Jämbeck, J. P. M.; Lyubartsev, A. P. Derivation and Systematic Validation of a Refined All-Atom Force Field for Phosphatidylcholine Lipids. *J. Phys. Chem. B* **2012**, *116* (10), 3164–3179. https://doi.org/10.1021/jp212503e.

(18) Jämbeck, J. P. M.; Lyubartsev, A. P. An Extension and Further Validation of an All-Atomistic Force Field for Biological Membranes. *J. Chem. Theory Comput.* **2012**, *8* (8), 2938–2948. https://doi.org/10.1021/ct300342n.
